# Review of the fish parasitic genus *Ceratothoa* Dana, 1852 (Crustacea, Isopoda, Cymothoidae) from South Africa, including the description of two new species

**DOI:** 10.3897/zookeys.400.6878

**Published:** 2014-04-10

**Authors:** Kerry A. Hadfield, Niel L. Bruce, Nico J. Smit

**Affiliations:** 1 Department of Zoology, University of Johannesburg, P.O. Box 524, Auckland Park, 2006 South Africa; 2 Museum of Tropical Queensland, Queensland Museum and School of Marine and Tropical Biology, James Cook University; 70–102 Flinders Street, Townsville, Australia 4810; 3 Water Research Group (Ecology), Unit for Environmental Sciences and Management, Potchefstroom Campus, North West University, Private Bag X6001, Potchefstroom, 2520, South Africa

**Keywords:** Isopod, cymothoid, buccal-cavity, fish parasite, tongue-biter, Indian Ocean

## Abstract

The genus *Ceratothoa* Dana, 1852 is revised for South African waters and re-diagnosed. *Ceratothoa retusa* (Schioedte & Meinert, 1883) is recorded from the eastern coast, and *Ceratothoa africanae* **sp. n.** and *C. famosa* **sp. n.** are described; *C. imbricata* (Fabricius, 1775) and *C. trigonocephala* (Leach, 1818), are redescribed, revised and excluded from the South African fauna. *Ceratothoa africanae* **sp. n.** can be distinguished by the stout body shape of the female; triangular cephalon with a pointed rostrum; short uropods which do not extend past the pleotelson; large carinae on the pereopod basis; a broad pleon; and large medial lobes on female pleopods. *Ceratothoa famosa* **sp. n.** is characterised by the long rectangular body shape; pereonite 1 with a raised medial protrusion; narrow antenna with antennule article 1 expanded; uropods which reach the posterior margin of the pleotelson; narrow rami on uropods; and no appendix masculina on pleopod 2 of the male specimens.

## Introduction

Cymothoid isopods are obligate parasites of both freshwater and marine fishes, where they will attach to the external surfaces, gills or inside the buccal-cavity of their fish host (Kensley and Schotte 1989, Trilles 1991). These isopods are economically important parasites as they have been shown to cause detrimental effects on fish in captivity including growth inhibition, malnutrition, anaemia and death in smaller fish (Romestand 1979, Bragoni et al. 1984, Adlard and Lester 1994, Horton and Okamura 2001, Mladineo 2002, Ravi and Rajkumar 2007). One of the most common genera of tongue-biters (cymothoids found inside the buccal-cavity of the fish host, attached to the tongue) in southern Africa is *Ceratothoa* Dana, 1852.

Very little is known about the cymothoid isopods from southern Africa and the western Indian Ocean (Kensley 1978, 2001). Recently, two buccal attaching genera have been reviewed; Hadfield et al. (2010) revised the monotypic genus *Cinusa* Schioedte & Meinert, 1884, endemic to this region and Hadfield et al. (2013) reviewed *Cymothoa* Fabricius, 1787 from the southwestern Indian Ocean.

*Ceratothoa* has long been considered to have three species in South Africa: *Ceratothoa imbricata* (Fabricius, 1775), *Ceratothoa retusa* (Schioedte & Meinert, 1883) and *Ceratothoa trigonocephala* (Leach, 1818) (see Kensley 2001). In the present study, none of the material agreed with the descriptions of *Ceratothoa imbricata* and *Ceratothoa trigonocephala*, and no positive identification for these two species in South Africa could be made. These species are therefore excluded from the South African fauna. However, sampling revealed two new species from the region, leaving the total at three species of *Ceratothoa* in South Africa.

## Methods

Type material for *Ceratothoa imbricata* and *Ceratothoa trigonocephala* were borrowed from the Natural History Museum, UK. All available material from Iziko South African Museum labelled as a *Ceratothoa* species was borrowed with additional specimens being obtained from fish hosts held in the South African Institute for Aquatic Biodiversity (SAIAB), Grahamstown, South Africa.

New material was collected along the south coast of South Africa by the FRS *Africana* and from intertidal rock pools at Tsitsikamma National Park.

Isopods were processed according to the techniques described in Hadfield et al. (2010, 2011, 2013). Species descriptions were prepared in DELTA (Descriptive Language for Taxonomy, see Coleman et al. 2010) using a general Cymothoidae character set. Classification follows Brandt and Poore (2003).

Host nomenclature and distribution are from FishBase (Froese and Pauly 2013).

Synonymies: Those records that we have been unable to confirm directly from specimens or the published figures, or that we otherwise have reasonable doubts about, have been removed from the synonymy.

**Abbreviations.** BMNH—British Museum, Natural History, UK (now NHMUK); MNHN—Muséum National d’Histoire Naturelle, Paris; NHMUK—Natural History Museum, UK; SAIAB—South African Institute for Aquatic Biodiversity, Grahamstown; SAM—South African Museum, Cape Town; SMNH—Swedish Museum of Natural History, Stockholm; ZMUC—Zoological Museum, University of Copenhagen; TL—total length; W—width.

## Taxonomy

### Family Cymothoidae Leach, 1814

#### Genus Ceratothoa

Dana, 1852

http://species-id.net/wiki/Ceratothoa

Ceratothoa Dana, 1852: 303; Miers 1876: 104–105; Haswell 1882: 282; Schioedte and Meinert 1883: 322–323; Richardson 1905: 233–234; Bowman 1978: 217–218; Brusca 1981: 177–178; Bruce and Bowman 1989: 1–2; Horton 2000: 1041; Martin et al. 2013: 396.
Codonophilus Haswell, 1881: 471.– 1882: 283; Hale 1926: 201, 223.
Rhexana Schioedte & Meinert, 1883: 289–290.
Cteatessa Schioedte & Meinert, 1883: 296–297.
Meinertia Stebbing, 1893: 354.– 1900: 642; 1910a: 103; Richardson 1905: 236–237; Menzies 1962: 116; Schultz 1969: 156.
Rhexanella Stebbing, 1911: 179.
Ceratothoa (Not).– Dana 1853: 747; Richardson 1905: 236; Schultz 1969: 155; Kussakin 1979: 287 [= *Glossobius* Schioedte & Meinert, 1883].

##### Type species.

Dana (1852) included two species, *Cymothoa gaudichaudii* Milne Edwards, 1840 and *Cymothoa parallela* Otto, 1828 in his new genus without designating a type species (Bowman 1978). Bowman (1978) resolved the generic name, concluding that *Ceratothoa* had priority over other names that had been in use, but did not designate a type species. The whereabouts of the *Cymothoa parallela* type specimen is unknown, and is thought to no longer be extant (Bruce and Bowman 1989, Horton 2000, Martin et al. 2013). Horton (2000), however, designated a neotype of *Cymothoa parallela* from Oran (but without a redescription) after Schioedte and Meinert (1883) referred to it as a "specim. typ.". The syntype female for *Cymothoa gaudichaudii* is in pieces (the male syntype is still intact) and held at the Muséum National d’Histoire Naturelle, Paris (Trilles 1973, Hadfield pers. obs.). A type species should ideally be designated only when one or both of the species is fully redescribed and its identity and type material clearly established.

##### Diagnosis.

Body narrow, strongly vaulted, 2.1–2.9 times as long as wide, widest at pereonite 5. Cephalon triangular, with blunt rostrum, anterior margin ventrally directed, posterior margin straight. Antennular bases in contact, broad and expanded, subequal to antenna. Eyes distinct. Mandible not expanded; mandible palp article 2 longer than article 3. Maxilla medial lobe partly fused, prominent nodulose spines on each lobe. Maxillule with 4 terminal spines. Maxilliped article 3 with 2 recurved spines, with oostegite lobe. Pereonite 1 anterolateral angles extensions encompassing cephalon. Pereonites 6 and 7 posterolateral margins not produced. Pereonite 7 extends past pleonite 1. Pleon subequal or narrower than pereon. Pleonite 1 width narrower than other pleonites, pleonites 2–5 subequal in width. Pleotelson narrower than pleonites. Coxae 5–7 visible, reniform, often produced and rounded, shorter than somite. Brood pouch from coxae 1–4 and 6, posterior pocket absent. Pereopods 5–7 basis with large blade-like carina, without robust setae. Pereopod 7 slightly larger or more than 1.5 times longer than pereopod 1. Pleopods from dorsal view not visible, decreasing in size posteriorly. Pleopods 1–5 with small pleats or pockets, with proximomedial lamellar lobe (more pronounced in pleopods 3–5), peduncle lobes on the lateral margin absent. Uropod rami short, not extending past posterior margin of pleotelson, subequal.

##### Remarks.

*Ceratothoa* can best be identified by the triangular cephalon, contiguous antennular bases, pleonite 1 narrower than the other pleonites, elongate body (2.1–2.9 times as long as wide), and subequal uropod rami which extend to the posterior margin of the pleotelson. Bruce and Bowman (1989) highlighted that *Ceratothoa* has unique pereopod morphology, with most species having prominent expansions on the basis of the posterior pereopods (pronounced carina), except *Ceratothoa gilberti* (Richardson, 1904) that has no expansions on any of the pereopods. Furthermore, the ischium of the posterior pereopods is also expanded in some species such as *Ceratothoa guttata* (Richardson, 1910) (see Bruce and Bowman 1989) and *Ceratothoa carinata* (Bianconi, 1869) (see Martin et al. 2013).

The most recent reviews of this genus are those of Bruce and Bowman (1989) and Martin et al. (2013). *Meinertia* Stebbing, 1893 and *Codonophilus* Haswell, 1881 were placed into synonymy with *Ceratothoa*, the senior available name by Bowman (1978), and Bruce and Bowman (1989) synonymised *Cteatessa* Schioedte & Meinert, 1883 and *Rhexanella* Stebbing, 1911 with *Ceratothoa*. *Glossobius* is distinct from *Ceratothoa* and is considered a valid genus which includes species associated with pelagic beloniform fishes (Exocoetidae, Hemirhamphidae).

##### Relationships.

Phylogenetic relationships of the cymothoid genera remain unassessed, other than comments given by Brusca (1981), Bruce and Bowman (1989), and the molecular analyses (using small data sets) of Ketmaier et al. (2008) and Jones et al. (2008). Brusca (1981) postulated that there were three evolutionary “lineages” within Cymothoidae based on their attachment sites on the hosts (external surfaces, buccal+gill, and the freshwater flesh burrowing genera). Both Ketmaier et al. (2008) and Jones et al. (2008) later demonstrated that these lineages could not necessarily be upheld.

In a preliminary phylogenetic analysis using 23 cymothoid genera, with *Rocinela* Leach, 1818 (Aegidae) as the outgroup (Hadfield 2012), the buccal-cavity isopods grouped together in a clade based on these genera having a cephalon encompassed by the anterolateral margins of pereonite 1, pereopods 5–7 with a large blade-like carina on the basis, and a partly fused maxilla medial lobe (with the exception of *Glossobius* which has lobed anterolateral margins and a distinct maxilla mesial lobe and *Lobothorax* Bleeker, 1857 which has no carina on the basis).

*Ceratothoa* is most closely related to *Glossobius* and this was shown in the preliminary study where the two genera grouped as sister taxa (Hadfield 2012). These genera share many similar characteristics such as the antennular bases being in contact (the apomorphic character for this clade); expanded antennules; antennules subequal to antennae; maxilliped article 3 with 2 recurved spines; and no peduncle lobes on the pleopods. *Ceratothoa* is distinguished from *Glossobius* by having distinct eyes; maxilla medial lobe partly fused rather than distinct; maxilliped with only one oostegite lobe compared to the two in *Glossobius*; anterolateral margins of pereonite 1 extended (not lobed as in *Glossobius*); and uropod rami are subequal.

#### Ceratothoa retusa

(Schioedte & Meinert, 1883)

http://species-id.net/wiki/Ceratothoa_retusa

Ceratothoæ retusæ Schioedte & Meinert in Hilgendorf 1879: 847 [nomen nudum].
Cteatessa retusa . – Schioedte & Meinert, 1883: 297–299, tab. XI (Cym. XVIII) Figs 11–13; Stebbing 1908: 424; Barnard 1925: 393.– 1940: 491; Nierstrasz 1931: 131; Trilles 1986: 625, tab. 1; 1994: 130; 2008: 23; Kensley 1978: 79–80, Figs 32 (g–h).
Codonophilus hemiramphi Pillai, 1954: 14–15 [nomen dubium].
Ceratothoa hemiramphi . – Trilles 1994: 120; Kensley 2001: 232.
Ceratothoa retusa . – Bruce and Bowman 1989: 8–12, Figs 5–8; Kensley 2001: 232; Trilles et al. 2011: 446–459; Hadfield et al. in press.

##### Distribution.

Indian Ocean—records from Mozambique, South Africa, Red Sea, India, Indonesia and northern Australia (see Hadfield et al. in press).

##### Hosts.

Hemirhamphidae buccal-cavity—*Hemirhamphus far* (Forsskål, 1775) and *Hemirhamphus robustus* Günther, 1866 (see Hadfield et al. in press).

##### Remarks.

*Ceratothoa retusa* can be identified by the large pereonite 1 with an anterolateral ridge and small cephalon sunken into pereonite 1. Pereonite 1 is deeply concave with anterolateral margins which almost extend to the tip of the cephalon. The pleotelson is broader than long and the uropods extend past the pleotelson margin (Hadfield et al. in press). This species was redescribed in detail by Hadfield et al. (in press) and has been shown to have a variable morphology depending on the sampling location of the specimen.

#### Ceratothoa africanae sp. n.

http://zoobank.org/B4BA5F68-2070-4464-88FB-B27356761920

http://species-id.net/wiki/Ceratothoa_africanae

**Figure 1.**
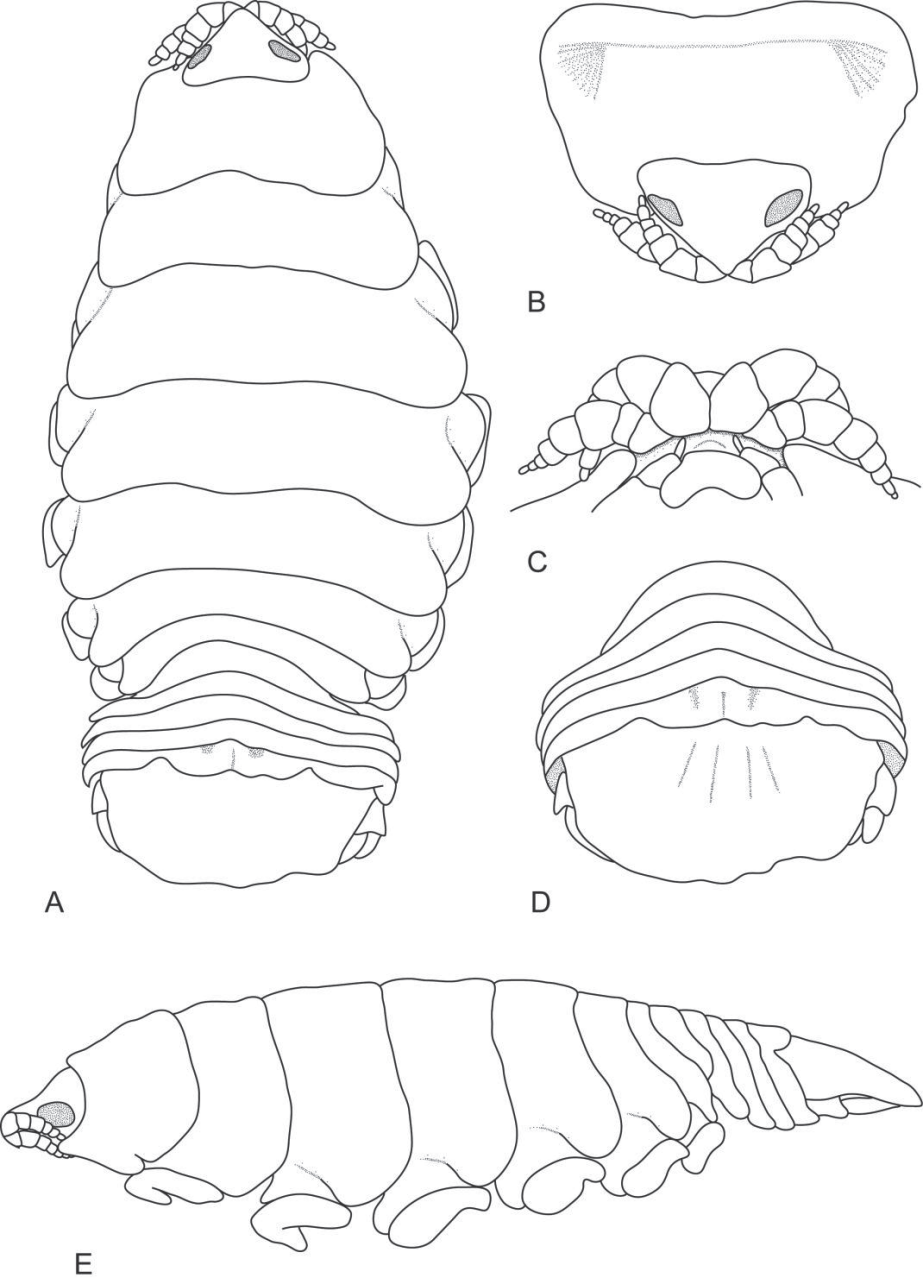
*Ceratothoa africanae* sp. n. female holotype (29 mm) (SAM A45937): **A** dorsal view **B** antero-dorsal view of pereonite 1 and cephalon **C** ventral view of cephalon **D** dorsal view of pleotelson **E** lateral view.

Figs 1 [Fig F2] [Fig F3] [Fig F4] [Fig F5] [Fig F6] –7 , 21

##### Material examined.

Holotype. Female (29 mm TL; 15 mm W), collected from a trawl (34°26'S, 24°13'E) along the south coast of South Africa from the buccal-cavity of *Spondyliosoma emarginatum*, 30-04-2003, coll. N.J. Smit (SAM A45937; HP 221).

Paratypes. All from the buccal-cavity of *Spondyliosoma emarginatum* and collected while trawling (34°26'S, 24°13'E) along the south coast of South Africa (30-04-2003), coll. N.J. Smit: Three females (22–26 mm TL; 12–15 mm W), three males (11–13 mm TL; 5–6 mm W), one dissected female (30 mm TL; 15 mm W), one dissected male (16 mm TL; 7 mm W) (SAM A45938; HP 221).

Other material. In the possession of authors at NWU. From the the buccal-cavity of *Spondyliosoma emarginatum*: Algoa Bay (33°51'S, 25°52'E), 1978: female (28 mm TL; 12 mm W). Eastern Cape, no date: female (20 mm TL; 11 mm W), male (7 mm TL; 3 mm W). Tsitsikamma Grootbank, Sout River (34°1'S, 23°28'E), September 1993: female (19 mm TL; 8 mm W).

**Figure 2. F2:**
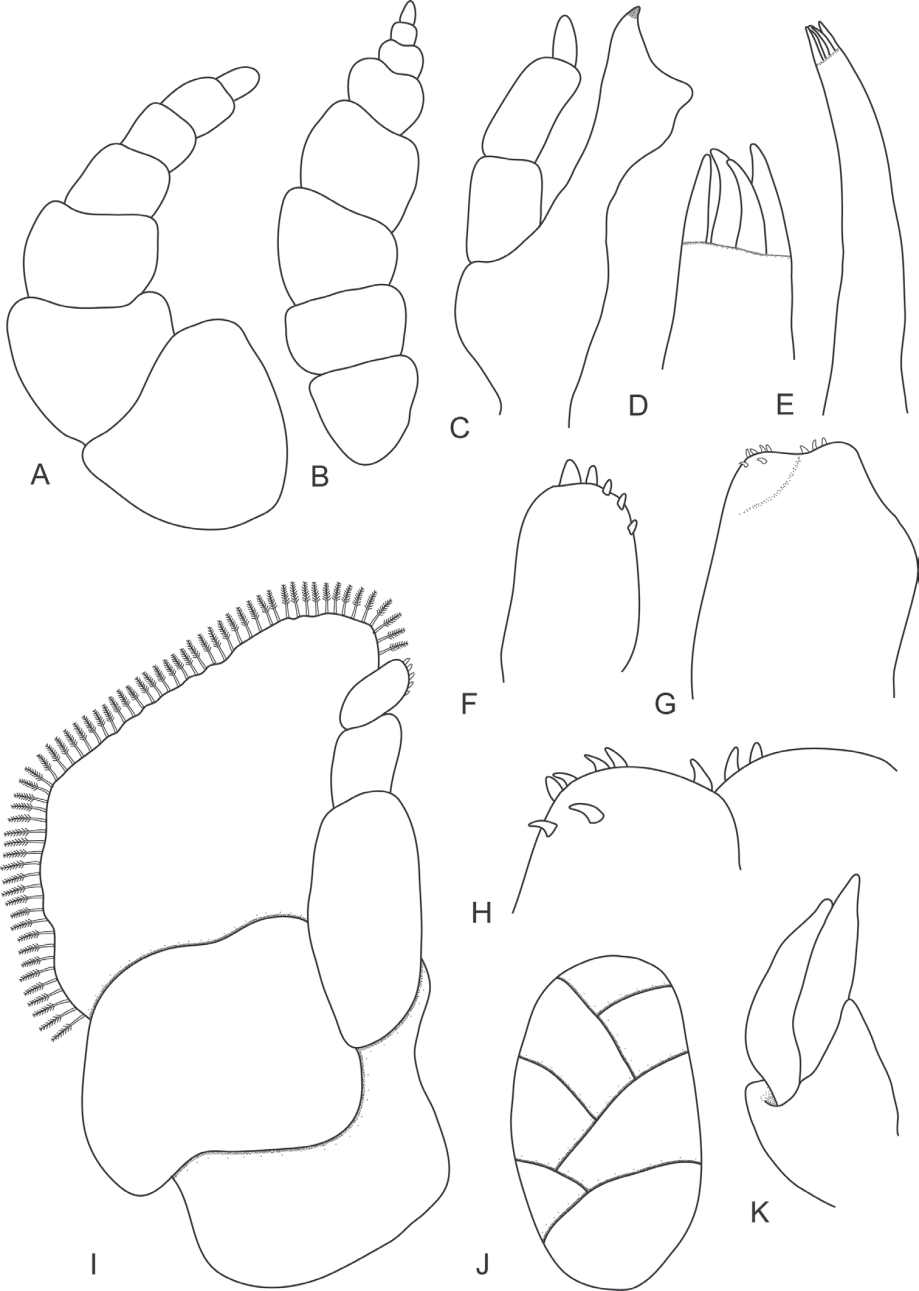
*Ceratothoa africanae* sp. n. female paratype (30 mm) (SAM A45938): **A** antennule **B** antenna **C** mandible **D** tip of maxillule **E** maxillule **F** tip of maxilliped article 3 **G** maxilla **H** tip of maxilla **I** maxilliped with oostegite **J** oostegites **K** uropod.

**Figure 3. F3:**
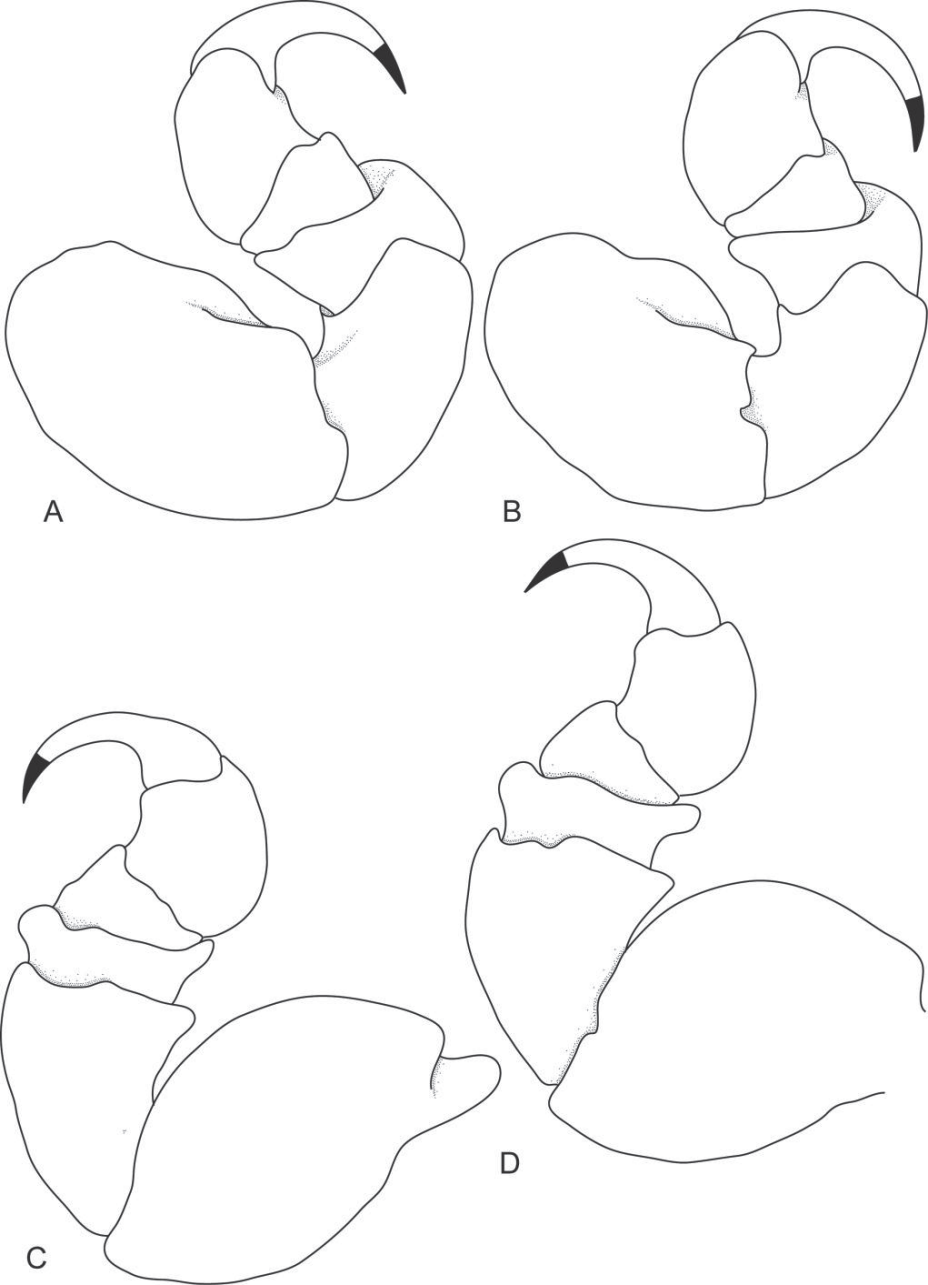
*Ceratothoa africanae* sp. n. female holotype (29 mm) (SAM A45937): **A** pereopod 1 **B** pereopod 2 **C** pereopod 6 **D** pereopod 7.

**Figure 4. F4:**
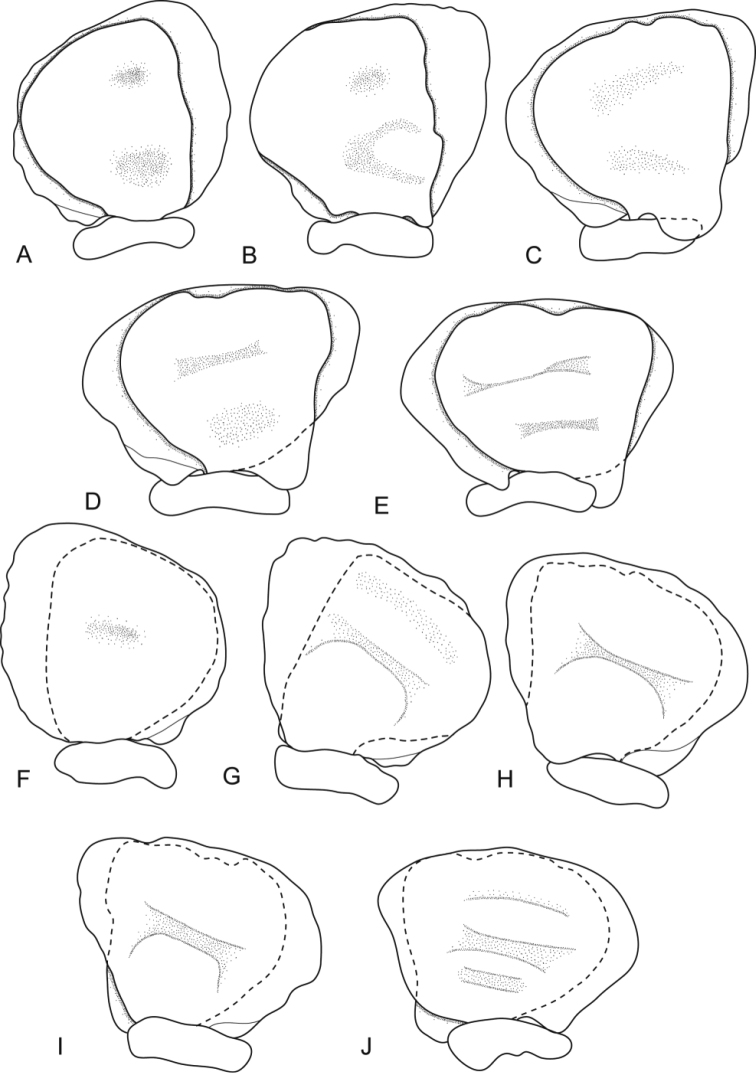
*Ceratothoa africanae* sp. n. female paratype (30 mm) (SAM A45938): **A** dorsal pleopod 1 **B** dorsal pleopod 2 **C** dorsal pleopod 3 **D** dorsal pleopod 4 **E** dorsal pleopod 5 **F** ventral pleopod 1 **G** ventral pleopod 2 **H** ventral pleopod 3 **I** ventral pleopod 4 **J** ventral pleopod 5.

**Figure 5. F5:**
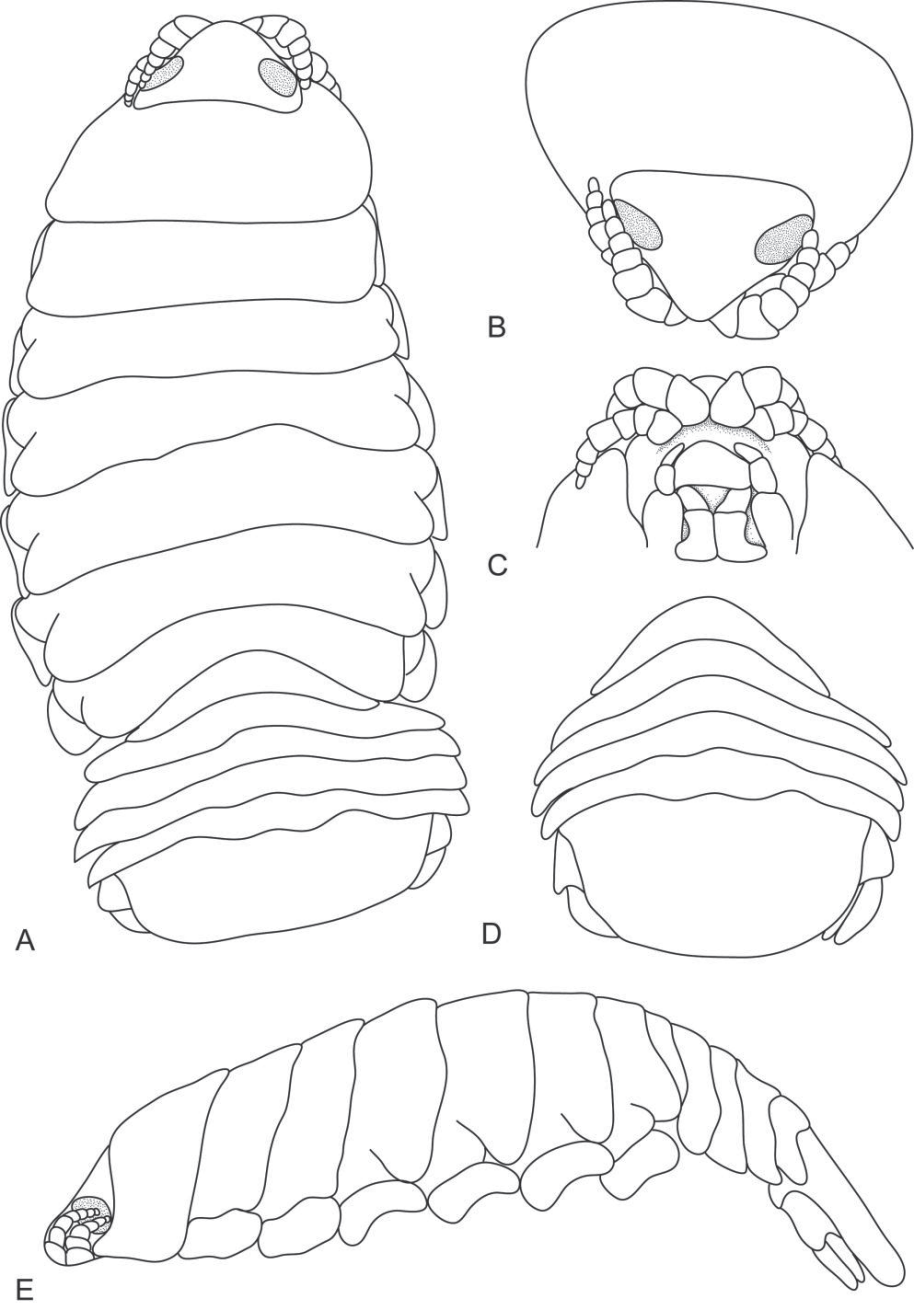
*Ceratothoa africanae* sp. n. male paratype (14 mm) (SAM A45938): **A** dorsal view **B** antero-dorsal view of pereonite 1 and cephalon **C** ventral view of cephalon **D** dorsal view of pleotelson **E** lateral view.

**Figure 6. F6:**
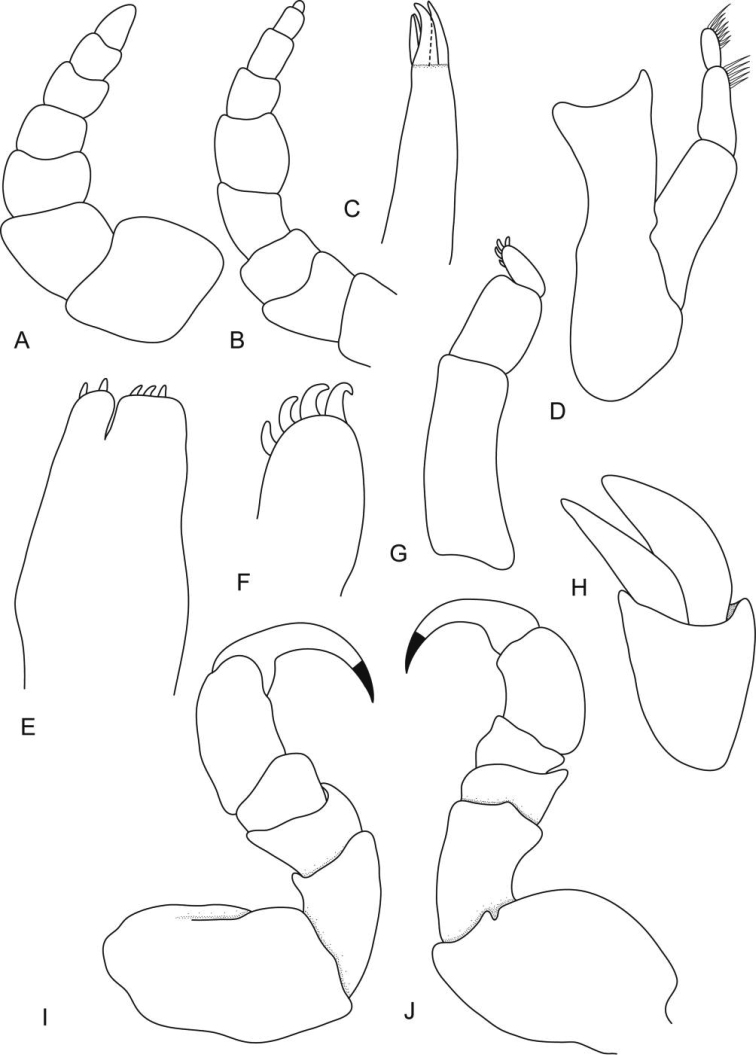
*Ceratothoa africanae* sp. n. male paratype (14 mm) (SAM A45938): **A** antennule **B** antenna **C** maxillule **D** mandible **E** maxilla **F** tip of maxilliped **G** maxilliped **H** uropod **I** pereopod 1 **J** pereopod 7.

**Figure 7.**
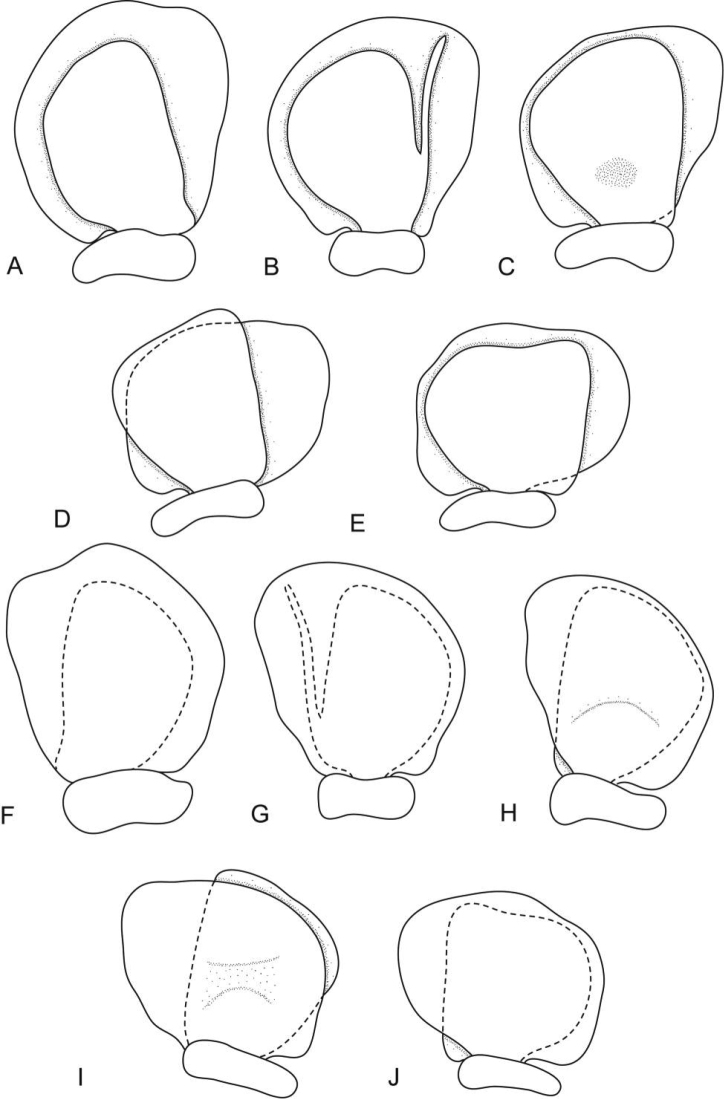
*Ceratothoa africanae* sp. n. male paratype (14 mm) (SAM A45938): **A** dorsal pleopod 1 **B** dorsal pleopod 2 **C** dorsal pleopod 3 **D** dorsal pleopod 4 **E** dorsal pleopod 5 **F** ventral pleopod 1 **G** ventral pleopod 2 **H** ventral pleopod 3 **I** ventral pleopod 4 **J** ventral pleopod 5.

##### Ovigerous female holotype.

Length 19–29 (23.4) mm, width 8–15 (12.5) mm.

Body ovoid, 1.5 times as long as greatest width, dorsal surfaces smooth and polished in appearance, widest at pereonite 4, most narrow at pereonite 1, lateral margins posteriorly ovate. Cephalon 0.6 times longer than wide, visible from dorsal view, triangular. Frontal margin rounded to form blunt rostrum. Eyes oval with distinct margins. Pereonite 1 with slight indentations, anterior border straight, anterolateral angle with small distinct anterior projection which does not extend past the eyes, posterior margins of pereonites smooth and straight. Coxae 2–3 with posteroventral angles not visible; 4–7 rounded. Pereonites 1–4 increasing in length and width; 5–7 decreasing in length and width; becoming more progressively rounded posteriorly. Pleon with pleonite 1 same width as other pleonites, visible in dorsal view; pleonites posterior margin smooth, mostly concave; posterolateral angles of pleonite 2 narrowly rounded, not posteriorly produced. Pleonites 3–5 similar in form to pleonite 2. Pleonite 5 with posterolateral angles free, not overlapped by lateral margins of pleonite 4, posterior margin with 2 indented points. Pleotelson 0.5 times as long as anterior width, dorsal surface smooth, lateral margins posteriorly narrow, posterior margin evenly rounded, without median point.

Antennule more stout than antenna, comprised of 7 articles; peduncle articles 1 and 2 distinct and articulated; article 2 0.9 times as long as article 1; article 3 0.3 times as long as combined lengths of articles 1 and 2, 0.7 times as long as wide; flagellum with 4 articles, extending to anterior of pereonite 1. Antenna comprised of 8 articles. Antenna peduncle article 3 1.5 times as long as article 2, as long as wide; article 4 0.8 times as long as wide, 0.8 times as long as article 3; article 5 0.5 times as long as article 4, 0.7 times as long as wide. Antenna flagellum with 3 articles, last article terminating in no setae, extending to anterior margin of pereonite 1. Anterior margin acute, with small median point. Mandibular process ending in an acute incisor, with no simple setae, mandible palp article 2 and 3 without setae. Maxillule simple with 4 terminal robust setae. Maxilla mesial lobe partly fused to lateral lobe; lateral lobe without simple setae, 3 recurved robust setae; mesial lobe with 6 large recurved robust setae and no simple setae. Maxilliped covered in pectinate scales and comprised of 3 articles, with lamellar oostegite lobe, palp article 2 without simple setae, article 3 with 5 recurved robust setae. Oostegites margin covered in numerous plumose setae, attached to pereopods 2–5.

Pereopod 1 basis 1.6 times as long as greatest width; ischium 0.7 times as long as basis; merus proximal margin with bulbous protrusion; carpus with straight proximal margin; propodus 1.3 times as long as wide; dactylus slender, 1.1 as long as propodus, 2.4 times as long as basal width. Pereopod 2 propodus 1.4 as long as wide; dactylus 1.1 as long as propodus. Pereopods gradually increasing in size towards posterior and all without robust or simple setae. Pereopod 6 basis 1.3 times as long as greatest width, ischium 0.9 times as long as basis, propodus 1.4 as long as wide, dactylus 1.3 as long as propodus. Pereopod 7 basis 1.3 times as long as greatest width; ischium 0.7 as long as basis, without protrusions; merus proximal margin with slight bulbous protrusion, merus 0.3 as long as ischium, 0.4 times as long as wide; carpus 1.3 as long as ischium, without bulbous protrusion, 0.8 times as long as wide; propodus 0.7 as long as ischium, 1.4 times as long as wide; dactylus slender, 1.3 as long as propodus, 3 times as long as basal width.

Pleopods without setae, exopod larger than endopod. Pleopod 1 exopod as long as wide, lateral margin strongly convex, distally truncate, mesial margin weakly convex; endopod 1.2 times as long as wide, lateral margin convex, distally subtruncate, mesial margin straight; peduncle 3.3 times as wide as long, without retinaculae. Pleopods 2–5 similar to pleopod 1 and mesial margins becoming more strongly produced. Pleopods 3–5 endopods proximal borders extending below exopod to peduncle. Large medial lobes present and increasing in size from pleopod 1 to 5.

Uropod more than half the length of pleotelson, peduncle 0.7 times longer than rami, peduncle lateral margin without setae; rami not extending beyond pleotelson, marginal setae absent, apices narrowly rounded. Endopod apically slightly pointed, 3.8 times as long as greatest width, lateral margin straight, terminating without setae, mesial margin straight. Exopod not extending to end of endopod, 4.1 times as long as greatest width, apically rounded, lateral margin weakly convex, terminating with no setae, mesial margin straight.

##### Male.

Length 7–14 (10.6) mm, width 3–7 (4.9) mm.

Males similar to females but much smaller. Body oval, 1.4 times as long as wide. Penis small, low tubercles. Pleopod 2 appendix masculina with parallel margins, 1.2 times as long as endopod, distally bluntly rounded.

##### Etymology.

Named for FRS *Africana*, from which the species was collected, also acknowledging that this is the first *Ceratothoa* species to be described from Africa.

##### Distribution.

Eastern Cape Province, South Africa: from Tsitsikamma to Algoa Bay.

##### Hosts.

Found in the buccal-cavity, on the tongue of *Spondyliosoma emarginatum* (Valenciennes, 1830).

##### Prevalence.

9/17 (53%) of *Spondyliosoma emarginatum* infected from the FRS *Africana* trawls, 4/68 (5.9%) from the SAIAB collections.

##### Remarks.

*Ceratothoa africanae* sp. n. can be distinguished by the stout body shape of the female; a pointed rostrum; short and stout antennae; uropods which do not extend past the pleotelson; a broad pleon; large medial lobes on female pleopods; and an appendix masculina on the second pleopod in male specimens. This species was compared to the known species from South Africa at the time (*Ceratothoa imbricata*, *Ceratothoa retusa* and *Ceratothoa trigonocephala*) and found to be distinct. Upon comparisons to other known species worldwide, it was concluded to be a new species.

*Ceratothoa africanae* sp. n. differs from *Ceratothoa retusa* in having a larger cephalon not sunken into pereonite 1 as seen in *Ceratothoa retusa*; lacks the anterolateral ridge on pereonite 1; has shorter uropods which do not extend past the posterior margin of the pleotelson; and lacks the large extended anterolateral margins on pereonite 1 which extend more than half the length of the cephalon in *Ceratothoa retusa* but less than half in *Ceratothoa africanae*.

*Ceratothoa africanae* sp. n. shares many similarities with *Ceratothoa imbricata* and *Ceratothoa famosa* sp. n. *Ceratothoa africanae* resembles *Ceratothoa imbricata* in having pereonite 1 longer than pereonites 2–4 and both have two concave mediolateral indents on the pleonite 5 posterior margin, but differs in having a broader body, anterolateral angles on pereonite 1 which do not extend past the eyes as is seen in *Ceratothoa imbricata*, shorter uropods that do not extend past the pleotelson and an acute cephalon anterior margin. The pleopods of *Ceratothoa africanae* have a few smaller lobes and folds and the pereopod 6 and 7 merus is produced on both the anterior and posterior sides. Furthermore, *Ceratothoa africanae* pereonite 7 does not overlap any pleonites and pereopods 1 to 3 have a smaller merus, but pereopods 4 to 7 are larger. *Ceratothoa africanae* and *Ceratothoa famosa* (see below) differ in the number of setae on the mandibular palp (five on *Ceratothoa africanae* and three on *Ceratothoa famosa*); no setae on the maxilliped palp in *Ceratothoa africanae* sp. n. but seven setae on *Ceratothoa famosa*; and nine setae on *Ceratothoa africanae* maxilla but ten on *Ceratothoa famosa*. More differences are noted in the remarks on *Ceratothoa famosa* sp. n.

#### Ceratothoa famosa sp. n.

http://zoobank.org/6F47F60D-9157-446F-9A2E-8A189549F087

http://species-id.net/wiki/Ceratothoa_famosa

**Figure 8.**
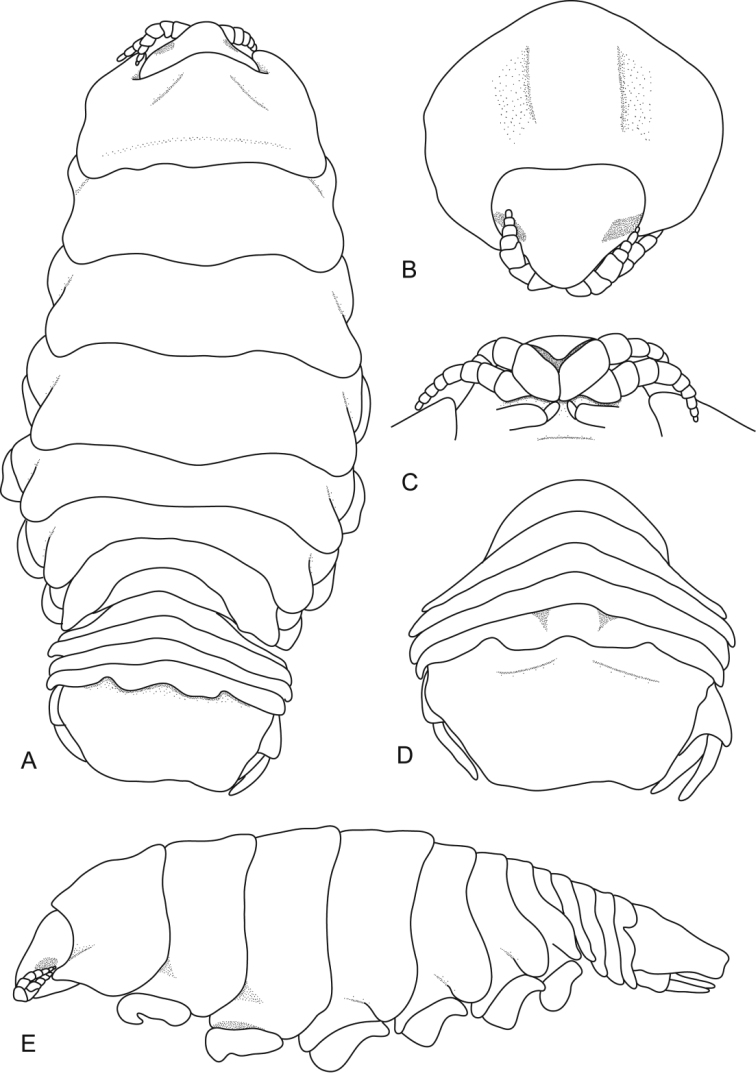
*Ceratothoa famosa* sp. n. female holotype (28 mm) (SAM A45939): **A** dorsal view **B** anterior view of pereonite 1 and cephalon **C** ventral view of cephalon **D** dorsal view of pleotelson **E** lateral view.

Figs 8 [Fig F9] [Fig F10] [Fig F11] [Fig F12] [Fig F13] –14 , 21

Meinertia imbricata ? .– Trilles 1972: 1248–1250, pl. II (10–11).

##### Material examined.

Holotype. Female (23 mm TL; 10 mm W), collected from Tsitsikamma National Park (34°1'S, 23°52'E) along the south coast of South Africa from the buccal-cavity of *Diplodus sargus capensis*, March 2005, coll. K.A. Hadfield (SAM A45939).

Paratypes. All from Tsitsikamma National Park (34°1'S, 23°52'E), Western Cape Province. From the buccal-cavity of *Diplodus sargus capensis*: dissected female (27 mm TL; 12 mm W), dissected male (13 mm TL; 6 mm W), April 2009, coll. K.A. Hadfield (SAM A45940); female (17 mm TL; 7 mm W), males (7, 14 mm TL; 3, 6 mm W), March 2005, coll. K.A. Hadfield (SAM A45941).

From the buccal-cavity of *Sparadon durbanensis*: female (15 mm TL; 6 W), male (5.5 mm TL; 2 mm W), April 2009, coll. K.A. Hadfield (SAM A45942).

Other material. In the possession of authors at NWU. From *Diplodus sargus capensis*: Cape Agulhas (34°49'S, 20°0'E): female (18 mm TL; 7 mm W), male (9 mm TL; 3 mm W). Kenton-on-sea (33°42'S, 26°41'E): female (14 mm TL; 5 mm W), male (5 mm TL; 2 mm W), May 1974. Morgan Bay (32°42'S, 28°20'E): two females (10, 12 mm TL; 3, 4 mm W), April 2003. Swartkops River Estuary (33°52'S, 25°38'E): female (10 mm TL; 3 mm W), male (6 mm TL; 2 mm W), July 1980. Transkei, between Goss Bay and Lupatana: female (10 mm TL; 4 mm W), September 1975. Transkei, Grosvenor Point (31°22'S, 29°53'E): female (15 mm TL; 6 mm W), male (6 mm TL; 2 mm W), September 1975. Tshani (31°56'S, 29°12'E): female (10 mm TL; 3 mm W), June 1996.

From *Diplodus cervinus hottentotus*: Kleinemonde (33°32'S, 27°03'E): female (25 mm TL; 6 mm W), male (5 mm TL; 2 mm W), June 1975; female (15 mm TL; 6 mm W), male (6 mm TL; 2 mm W), March 1975; Keiskamma River Mouth (33°16'S, 27°29'E): female (18 mm TL; 8 mm W), male (9 mm TL; 4 mm W), February 1976; Knysna (34°5'S, 23°3'E): female (19 mm TL; 7 mm W), male (8 mm TL; 3 mm W), 1945–1969. Tsitsikamma National Park (34°1'S, 23°52'E): female (20 mm TL; 7 W), male (14 mm TL; 5 mm W), 17 juveniles, March 2007.

From *Sparadon durbanensis*: Cape Padrone, Eastern Cape (33°46'S, 26°28'E): four pullus (5 mm TL; 2 mm W), July 1975. Kleinemonde (33°32'S, 27°03'E): five pullus (4 mm TL; 2 mm W), February 1977. Knysna (34°5'S, 23°3'E): two pullus (5 mm TL; 2 mm W), 1945–1965. Tsitsikamma National Park (34°1'S, 23°52'E): female (26 mm TL; 12 W), male (12 mm TL; 5 mm W), 35 juveniles, July 2008.

**Figure 9. F9:**
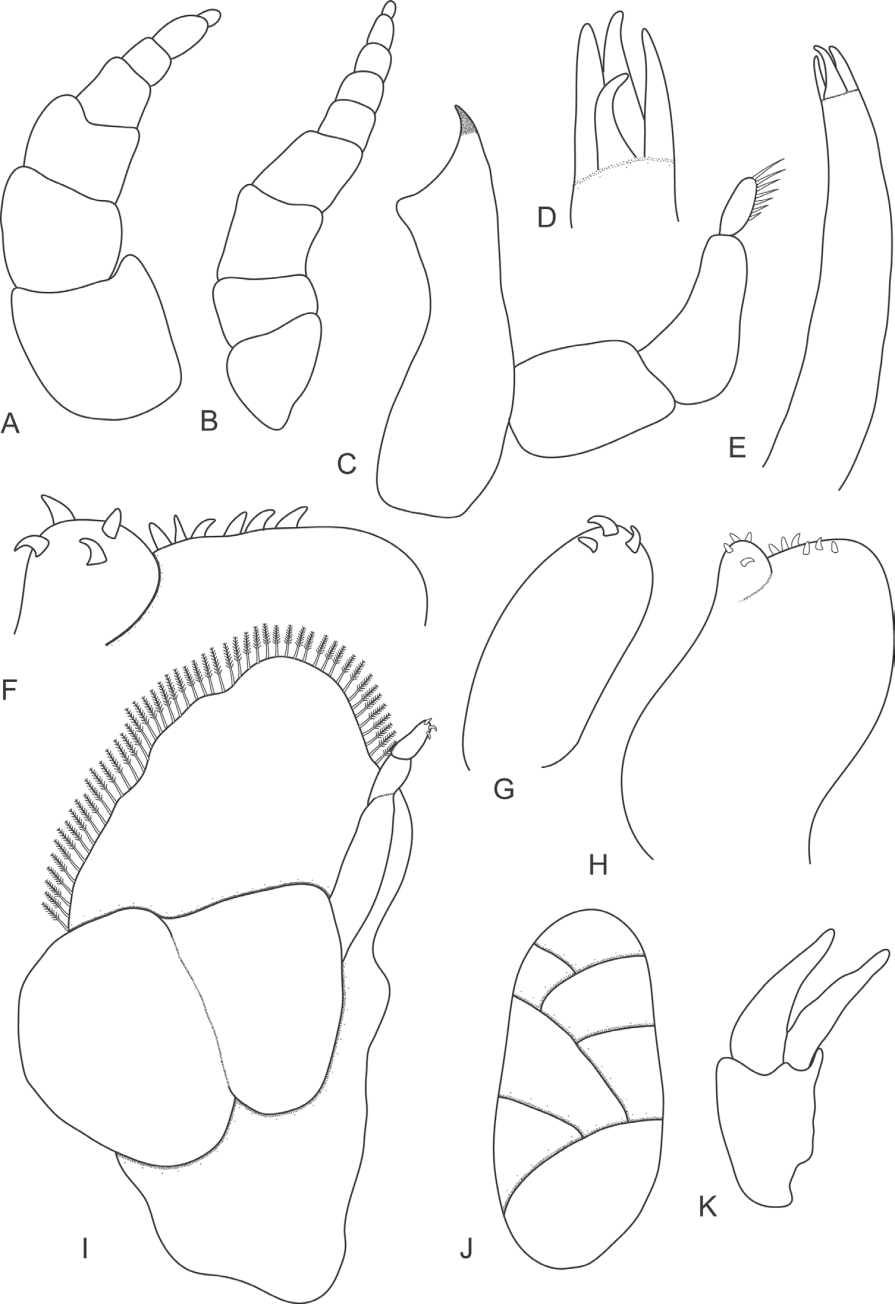
*Ceratothoa famosa* sp. n. female paratype (26 mm) (SAM A45941): **A** antennule **B** antenna **C** mandible **D** tip of maxillule **E** maxillule **F** tip of maxilla **G** tip of maxilliped article 3 **H** maxilla **I** maxilliped with oostegite **J** oostegites **K** uropod.

**Figure 10. F10:**
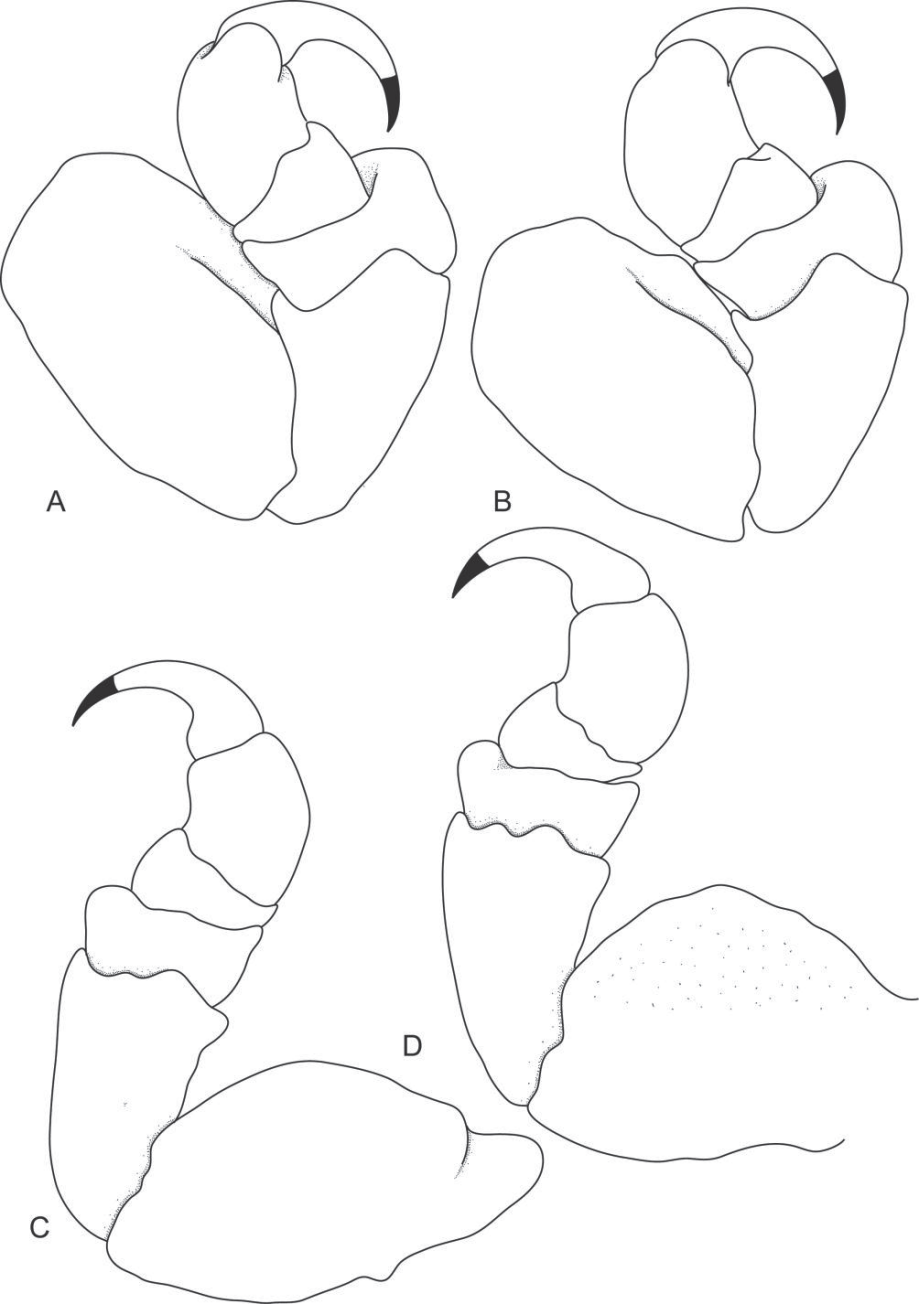
*Ceratothoa famosa* sp. n. female holotype (28 mm) (SAM A45939): **A** pereopod 1 **B** pereopod 2 **C** pereopod 6 **D** pereopod 7.

**Figure 11. F11:**
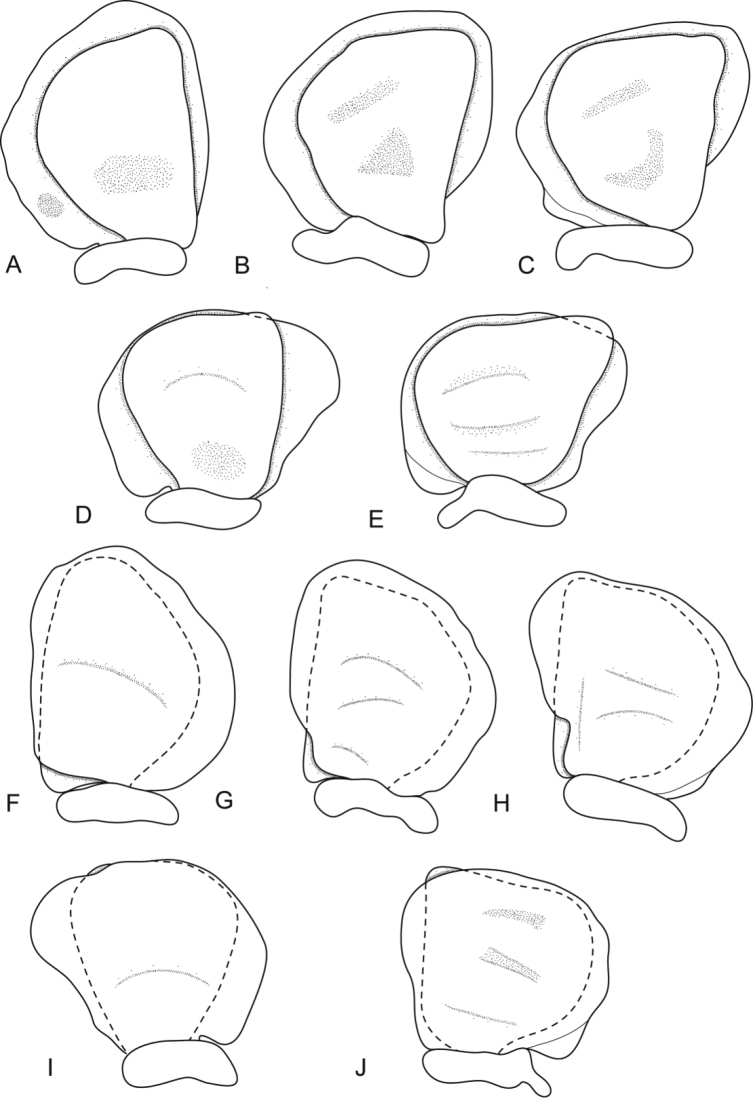
*Ceratothoa famosa* sp. n. female paratype (26 mm) (SAM A45941): **A** dorsal pleopod 1 **B** dorsal pleopod 2 **C** dorsal pleopod 3 **D** dorsal pleopod 4 **E** dorsal pleopod 5 **F** ventral pleopod 1 **G** ventral pleopod 2 **H** ventral pleopod 3 **I** ventral pleopod 4 **J** ventral pleopod 5.

**Figure 12. F12:**
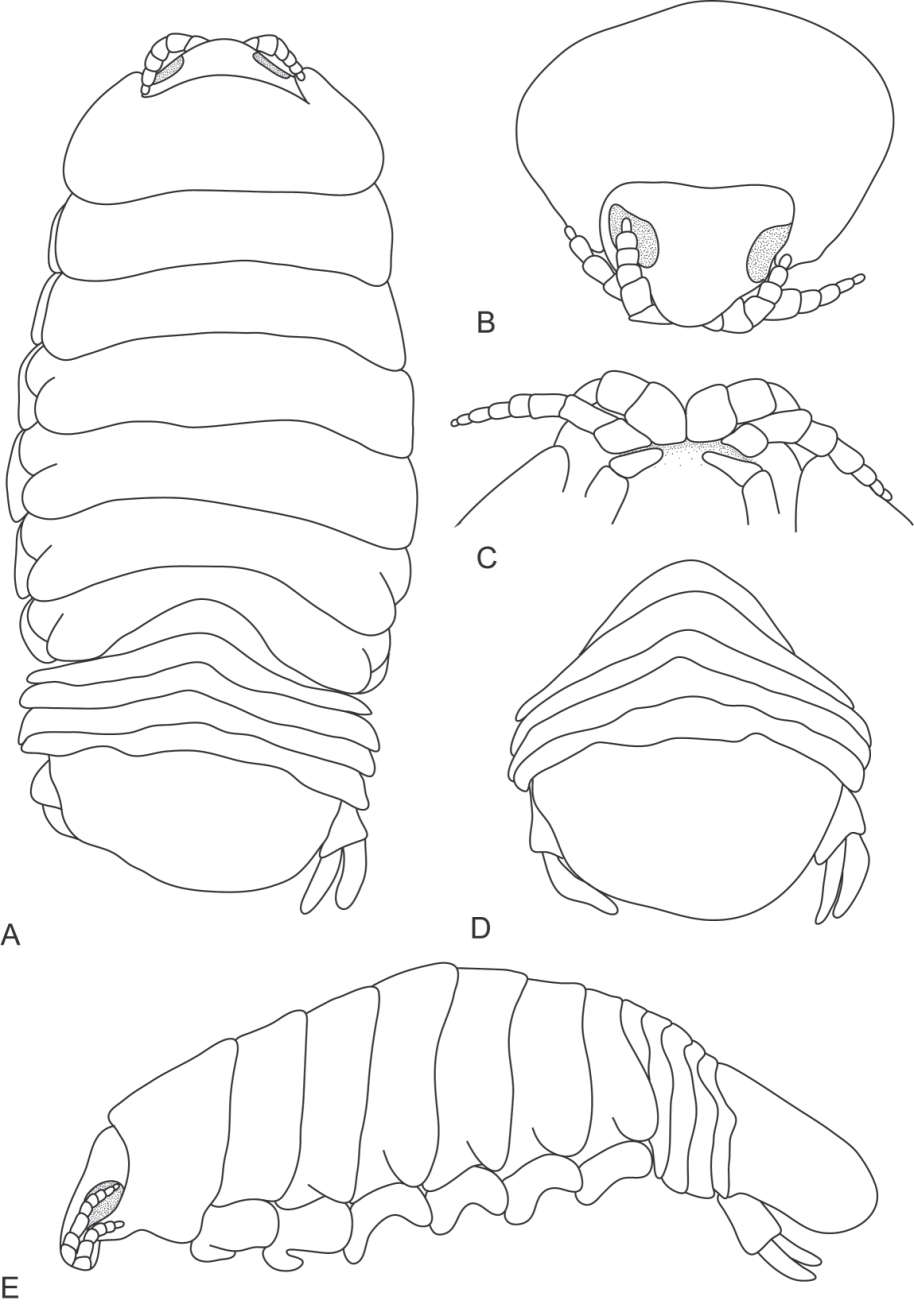
*Ceratothoa famosa* sp. n. male paratype (12 mm) (SAM A45941): **A** dorsal view **B** anterior view of pereonite 1 and cephalon **C** ventral view of cephalon **D** dorsal view of pleotelson **E** lateral view.

**Figure 13. F13:**
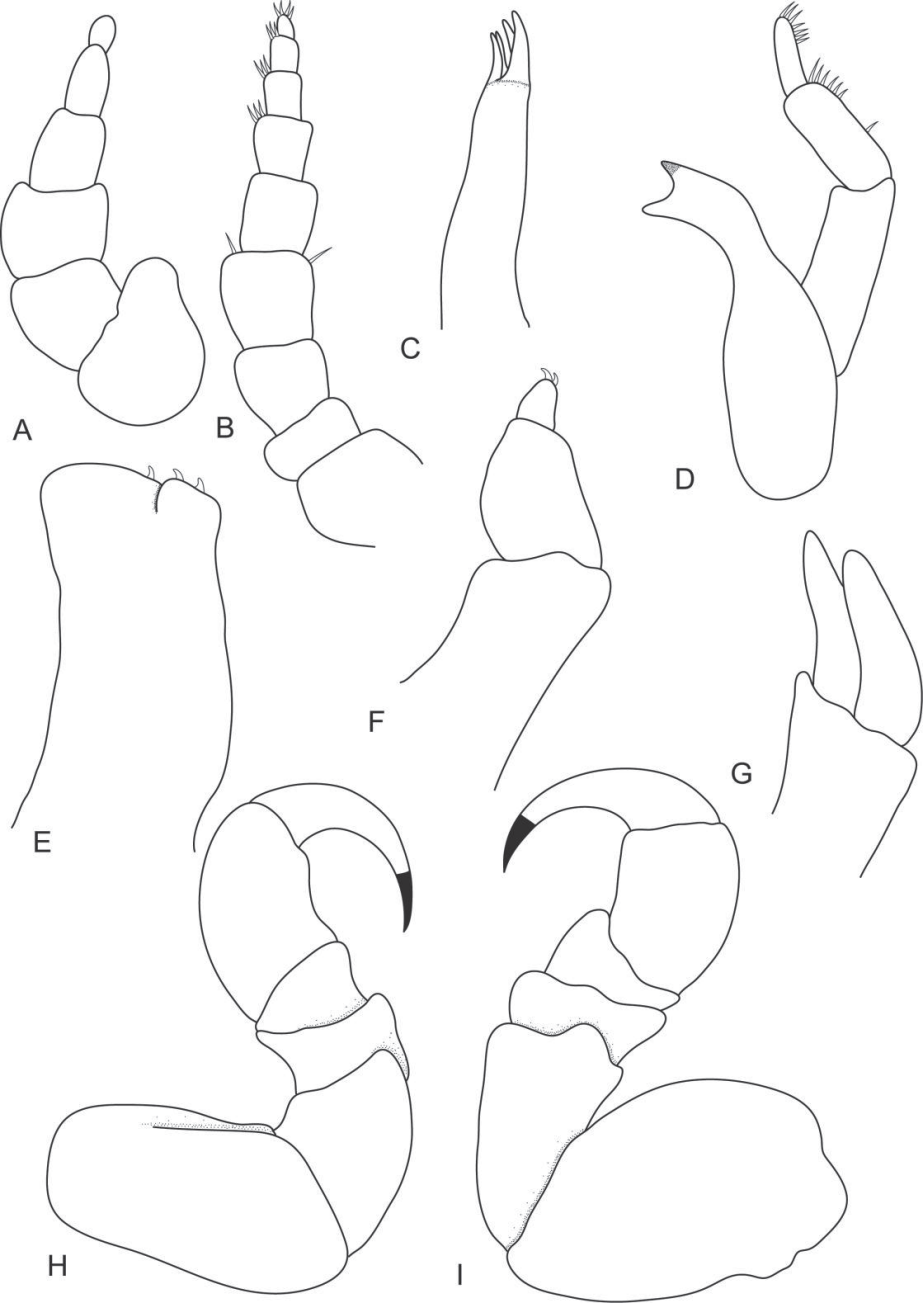
*Ceratothoa famosa* sp. n. male paratype (12 mm) (SAM A45941): **A** antennule **B** antenna **C** maxillule **D** mandible **E** maxilla **F** maxilliped **G** uropod **H** pereopod 1 **I** pereopod 7.

**Figure 14.**
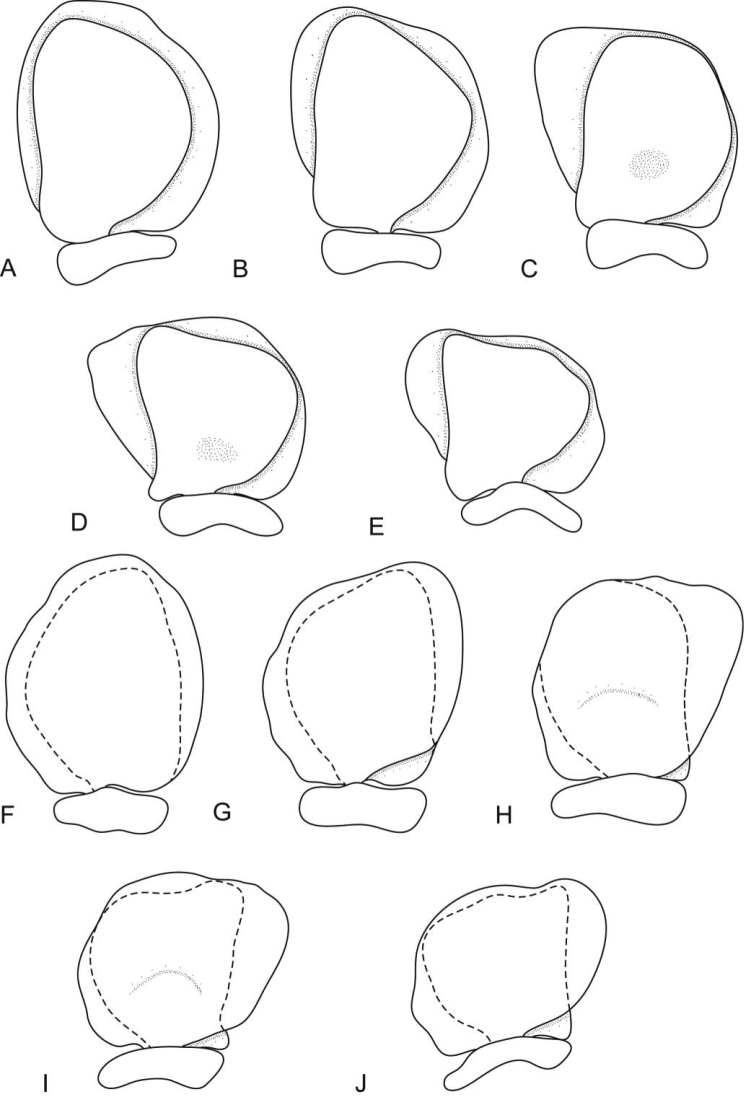
*Ceratothoa famosa* sp. n. male paratype (12 mm) (SAM A45941): **A** dorsal pleopod 1 **B** dorsal pleopod 2 **C** dorsal pleopod 3 **D** dorsal pleopod 4 **E** dorsal pleopod 5 **F** ventral pleopod 1 **G** ventral pleopod 2 **H** ventral pleopod 3 **I** ventral pleopod 4 **J** ventral pleopod 5.

##### Ovigerous female holotype.

Length 10–28 (16.9) mm, width 3–12 (6.0) mm.

Body rectangular, 1.7 times as long as greatest width, dorsal surfaces smooth and polished in appearance, widest at pereonite 4 and pereonite 5, most narrow at pereonite 7, lateral margins slightly convex. Cephalon 0.8 times longer than wide, slightly visible in dorsal view, triangular. Frontal margin rounded to form blunt rostrum. Eyes irregular in outline. Pereonite 1 with median projection, anterior border straight, anterolateral angle with distinct anterior projection, posterior margins of pereonites smooth and slightly curved laterally. Coxae 2–3 with posteroventral angles not visible; 4–7 rounded. Pereonites 1–5 increasing in length and width; 6–7 decreasing in length and width; becoming more progressively rounded posteriorly. Pleon with pleonite 1 same width as other pleonites, visible in dorsal view; pleonites posterior margin smooth, mostly concave; posterolateral angles of pleonite 2 narrowly rounded, not posteriorly produced. Pleonites 3–5 similar in form to pleonite 2. Pleonite 5 with posterolateral angles free, not overlapped by lateral margins of pleonite 4, posterior margin with 2 indented points and medial indent. Pleotelson 0.5 times as long as anterior width, dorsal surface smooth, lateral margins posteriorly narrow, posterior margin broadly truncate, without median point.

Antennule more stout than antenna, comprised of 7 articles; peduncle articles 1 and 2 distinct and articulated; article 2 0.8 times as long as article 1; article 3 0.3 times as long as combined lengths of articles 1 and 2, 0.8 times as long as wide; flagellum with 4 articles, extending to anterior of pereonite 1. Antenna comprised of 9 articles. Antenna peduncle article 3 1.4 times as long as article 2, 1.1 times as long as wide; article 4 1.1 times as long as wide, 0.9 times as long as article 3; article 5 0.5 times as long as article 4, 0.8 times as long as wide. Antenna flagellum with 4 articles, last article terminating in no setae, extending to anterior margin of pereonite 1. Anterior margin rounded, forming median point. Mandibular process ending in an acute incisor, with no simple setae, mandible palp article 2 with no distolateral setae, and article 3 with 7 serrate setae. Maxillule simple with 4 terminal robust setae. Maxilla mesial lobe partly fused to lateral lobe; lateral lobe with no simple setae, 6 recurved robust setae; mesial lobe with no simple setae, and 4 large recurved robust setae. Maxilliped weakly segmented, with lamellar oostegite lobe, palp article 2 with no simple setae, article 3 with 3 recurved robust setae, and no simple setae. Oostegites margin covered in numerous plumose setae, attached to pereopods 2–5.

Pereopod 1 basis 1.6 times as long as greatest width; ischium 0.8 times as long as basis; merus proximal margin with bulbous protrusion; carpus with straight proximal margin; propodus 1.4 times as long as wide; dactylus slender, 1.1 as long as propodus, 2.5 times as long as basal width. Pereopod 2 propodus 1.5 as long as wide; dactylus 1.1 as long as propodus. Pereopods gradually increasing in size towards posterior and all without robust or simple setae. Pereopod 6 basis 1.6 times as long as greatest width, ischium 0.8 times as long as basis, propodus 1.5 as long as wide, dactylus 1.2 as long as propodus. Pereopod 7 basis 1.3 times as long as greatest width; ischium 0.9 as long as basis, without protrusions; merus proximal margin with slight bulbous protrusion, merus 0.3 as long as ischium, 0.5 times as long as wide; carpus 1 as long as ischium, without bulbous protrusion, 0.7 times as long as wide; propodus 2.1 as long as ischium, 1.4 times as long as wide; dactylus slender, 1.3 as long as propodus, 2.9 times as long as basal width.

Pleopods without setae, exopod larger than endopod. Pleopod 1 exopod 1.1 times as long as wide, lateral margin weakly convex, distally broadly rounded, mesial margin straight; endopod 1.4 times as long as wide, lateral margin convex, distally subtruncate, mesial margin straight; peduncle 3.3 times as wide as long, without retinaculae. Pleopods 2–5 similar to pleopod 1. Pleopods 3–5 endopods proximal borders do not extend below exopod to peduncle. Large medial lobes absent.

Uropod same length as pleotelson, peduncle 1 times longer than rami, peduncle lateral margin without setae; rami extending to pleotelson apex, marginal setae absent, apices narrowly rounded. Endopod apically slightly pointed, 4.1 times as long as greatest width, lateral margin straight, terminating without setae, mesial margin straight. Exopod extending to end of endopod, 3.3 times as long as greatest width, apically rounded, lateral margin weakly convex, terminating with no setae, mesial margin straight.

##### Male.

Length 4–14 (8.2) mm, width 1–5 (3.3) mm.

Males similar to females but much smaller. Body rectangular, 1.6 times as long as wide. Penis small, low tubercles. Pleopod 2 appendix masculina absent.

##### Etymology.

A photograph by one of us (NJS) of this species in the mouth of a *Diplodus sargus capensis* from Tsitsikamma National park, posted on the internet in 2004 has been used in many media reports worldwide, including magazines, children’s books, documentaries, nature programmes, daily news reports, and even in a motion picture. The epithet is derived from *famosus* (Latin—famous) (Brown 1956).

##### Distribution.

Known from off the southern coast of South Africa: Cape Agulhas; Knysna; Tsitsikamma; Swartkops River Estuary; Kenton-on-sea; Kleinemonde; Keiskamma River Mouth; Morgan Bay; Tshani; Grosvenor Point; and Transkei (between Goss Bay and Lupatana).

##### Hosts.

Found on the tongue of *Diplodus sargus capensis* (Smith, 1844), *Diplodus cervinus hottentotus* (Smith, 1844) and *Sparadon durbanensis* (Castelnau, 1861).

##### Prevalence.

1/3 (33.3%) of *Diplodus cervinus hottentotus*, 6/20 (30%) of *Diplodus sargus capensis* and 6/33 (18.2%) of *Sparadon durbanensis* infected from Tsitsikamma National Park; 26/366 (7.1%) of *Diplodus cervinus hottentotus*, 78/1004 (7.8%) of *Diplodus sargus capensis* and 11/100 (11%) of *Sparadon durbanensis* from the SAIAB collections.

##### Remarks.

*Ceratothoa famosa* sp. n. can be distinguished by the long rectangular body shape, pereonite 1 with a raised medial protrusion, a blunt rostrum, narrow antenna with antennule article 1 expanded, uropods which reach the posterior margin of the pleotelson, pereopods 1 and 2 with large bulbous protrusion on merus, narrow rami on uropods, and no appendix masculina on pleopod 2 of the male specimens.

*Ceratothoa famosa* sp. n. is similar to *Ceratothoa trigonocephala* in having pereonites 1–4 almost subequal but has a more bluntly rounded anterior margin of the cephalon observed in *Ceratothoa imbricata* as well as the two mediolateral concave indents in pleonite 5. Specific characters for *Ceratothoa famosa* include an antennule with an enlarged first article; a medial protrusion on pereonite 1 creating a rounded elevation around the cephalon; and a rostral point which is folded over between the antennae. The anterolateral margins are close to the cephalon and are bluntly rounded extending just past the middle of the cephalon. The uropods are the same length as the pleotelson and the male specimens lack an appendix masculina on pleopod 2 as seen with *Ceratothoa oestroides* (Risso, 1826), *Ceratothoa italica* Schioedte & Meinert, 1883, *Ceratothoa capri* (Trilles, 1964c), *Ceratothoa gilberti* (Richardson, 1904) and *Cymothoa gaudichaudii*. The pleopods do not have many folds or lobes but the pereopods have large carinae and extended protrusions on the merus of pereopods 1 and 2.

Other differences between *Ceratothoa famosa* sp. n. and *Ceratothoa africanae* sp. n. is the rostral point, which is blunt and ventrally directed and does not fold over in *Ceratothoa africanae*.; *Ceratothoa famosa* pereonite 7 overlaps pleonite 1 and the P1–P4 merus has a large bulbous protrusion which is smaller in P5–P7 (opposite in *Ceratothoa africanae* sp. n.); and *Ceratothoa famosa*. has pointed rather than rounded anterolateral margins on pereonite 1 as seen in *Ceratothoa africanae*.

Miers (1876) commented that South African specimens in his possession from the Cape of Good Hope did not correspond to the specimens of *Ceratothoa imbricata* in the British Museum and that there was a probability that the specimens were a distinct species. It is probable that all the records of *Ceratothoa imbricata* (or *Ceratothoa banksii* Leach, 1818) from South Africa are *Ceratothoa famosa* sp. n.

### Excluded species

Two widely recorded species *Ceratothoa imbricata* (Fabricius, 1775) and *Ceratothoa trigonocephala* (Leach, 1818), both of which have long been considered to occur in South Africa (see Kensley 1978), are here excluded from the South African fauna. As there has been sustained confusion over the identity of these two species (see Miers 1884, Stebbing 1902, 1908, Nierstrasz 1915, Trilles 1973, Bruce et al. 2002), we present descriptions of the type material, and include differential remarks and brief comments on the distribution and host use for the species, based solely on those records we have been able to confirm. For a full synonymy and lists of all the host and locality records, see Trilles (1994) or Hadfield (2012).

#### Ceratothoa imbricata

(Fabricius, 1775)

http://species-id.net/wiki/Ceratothoa_imbricata

**Figure 15.**
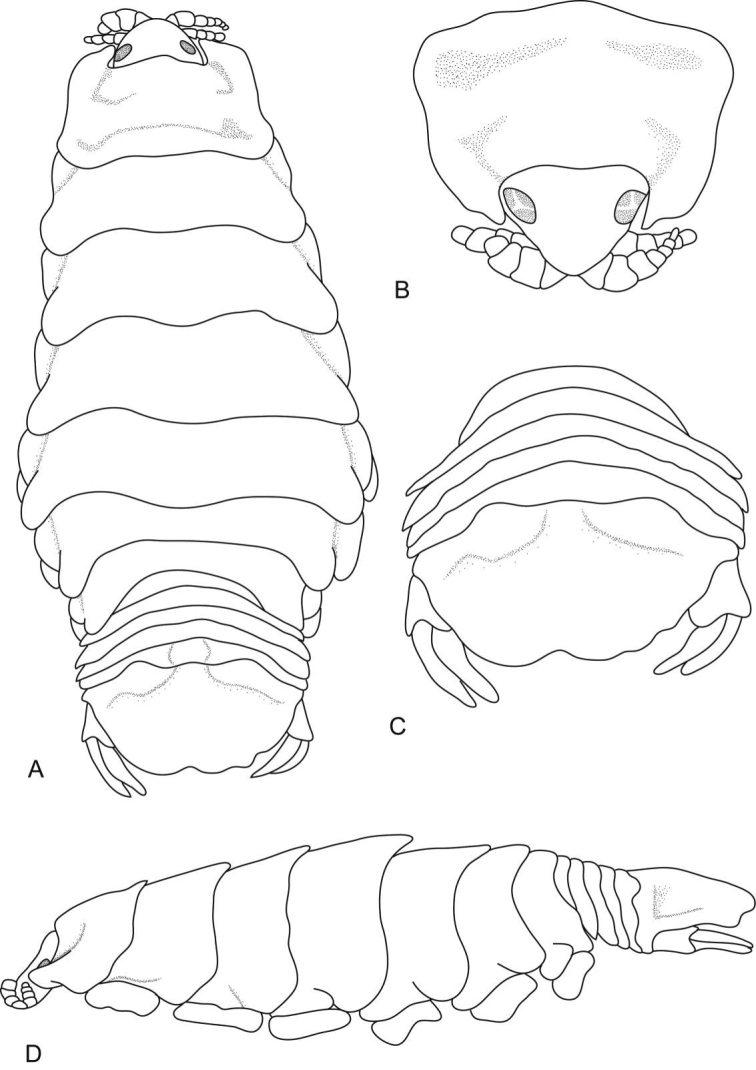
*Ceratothoa imbricata* (Fabricius, 1775), female holotype (34 mm) (BMNH 1979.403.1): **A** dorsal view **B** antero-dorsal view of pereonite 1 and cephalon **C** dorsal view of pleotelson **D** lateral view.

Figs 15 [Fig F16] [Fig F17] –18 , 21

Oniscus umbricatus Fabricius, 1775: 296.
Oniscus imbricatus . – Fabricius 1787: 241.
Cymothoa imbricata . – Fabricius 1793: 503; 1798: 304.
Cymothoa Banksii Leach, 1818: 353.
Ceratothoa Banksii . – Schioedte and Meinert 1883: 340–347, tab. XIV (Cym. XXI), Figs 6–21.
Ceratothoa imbricata . – Ellis 1981: 123.
Codonophilus imbricatus . – Hale 1926: 223–226, Figs 15–16; 1927: 315; 1929: 263–264, fig. 262; 1937: 19; 1940: 303.
Cymothoa banksii . – Ellis 1981: 124.

##### Material examined.

Holotype of *Ceratothoa imbricata*. The Natural History Museum, London (BMNH 1979.403.1) – female (34 mm TL; 16 mm W) collection of Sir Joseph Banks, Linnean Society, from New Zealand, coll. S.W.J. Banks, host unknown (Fabricius 1775). Noted: there is a hole in pereonite 4 and 5.

Holotype of *Ceratothoa banksii*. The Natural History Museum, London (BMNH 1979.402.1) – female (37 mm TL; 18 mm W), presented by Leach to the Museum of the Linnean Society, from New Zealand, White’s MS Cat No. 222, Coll. W.E. Leach, host unknown.

##### Description of holotype.

Body ovoid, 2.1 times as long as greatest width, dorsal surfaces slightly bumpy, widest at pereonite 5, most narrow at pereonite 1, lateral margins posteriorly ovate. Cephalon 0.7 times longer than wide, visible from dorsal view, triangular. Frontal margin rounded to form blunt rostrum. Eyes oval with distinct margins. Pereonite 1 with slight indentations, anterior border straight, anterolateral angle with distinct produced point extending to or beyond the eye margin, posterior margins of pereonites smooth and slightly curved laterally. Pereonites 1–5 increasing in length and width; 6–7 decreasing in length and width; 6 and 7 narrower. Pleon with pleonite 1 most narrow, visible in dorsal view; pleonites posterior margin smooth, mostly concave; posterolateral angles of pleonite 2 narrowly rounded, not posteriorly produced. Pleonites 3–5 similar in form to pleonite 2. Pleonite 5 with posterolateral angles free, not overlapped by lateral margins of pleonite 4, posterior margin produced medially. Pleotelson 2 times as long as anterior width, dorsal surface with lateral indent, lateral margins weakly convex, posterior margin rounded, without median point. Antennule more stout than antenna, comprised of 8 articles. Antenna comprised of 4 articles. Pereopod 1 basis 1.5 times as long as greatest width; ischium 0.8 times as long as basis; merus proximal margin with bulbous protrusion; carpus with straight proximal margin; propodus 1.5 times as long as wide; dactylus slender, 0.9 as long as propodus, 2.3 times as long as basal width. Pereopod 2 propodus 1.3 as long as wide; dactylus 0.6 as long as propodus. Pereopods 3 similar to pereopod 2. Pereopod 6 basis 1.4 times as long as greatest width, ischium 0.7 times as long as basis, propodus 1.5 as long as wide, dactylus 1 as long as propodus. Pereopod 7 basis 1.2 times as long as greatest width; ischium 0.7 as long as basis, without protrusions; merus proximal margin with slight bulbous protrusion, merus 0.4 as long as ischium, 0.6 times as long as wide; carpus 0.6 as long as ischium, without bulbous protrusion, 0.5 times as long as wide; propodus 0.6 as long as ischium, 1.4 times as long as wide; dactylus slender, 1.1 as long as propodus, 2.5 times as long as basal width. Uropod longer than the pleotelson, peduncle 0.7 times longer than rami, peduncle lateral margin without setae; rami extending beyond pleotelson, marginal setae absent, apices narrowly rounded.

**Figure 16. F16:**
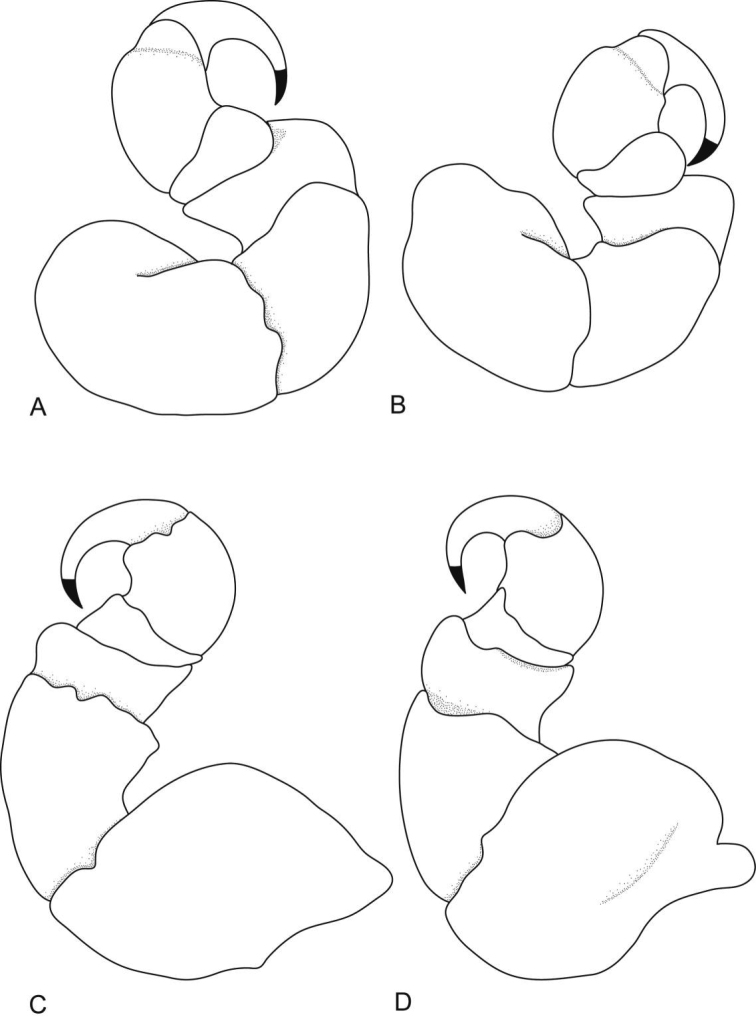
*Ceratothoa imbricata* (Fabricius, 1775), female holotype (34 mm) (BMNH 1979.403.1): **A** pereopod 1 **B** pereopod 2 **C** pereopod 6 **D** pereopod 7.

**Figure 17. F17:**
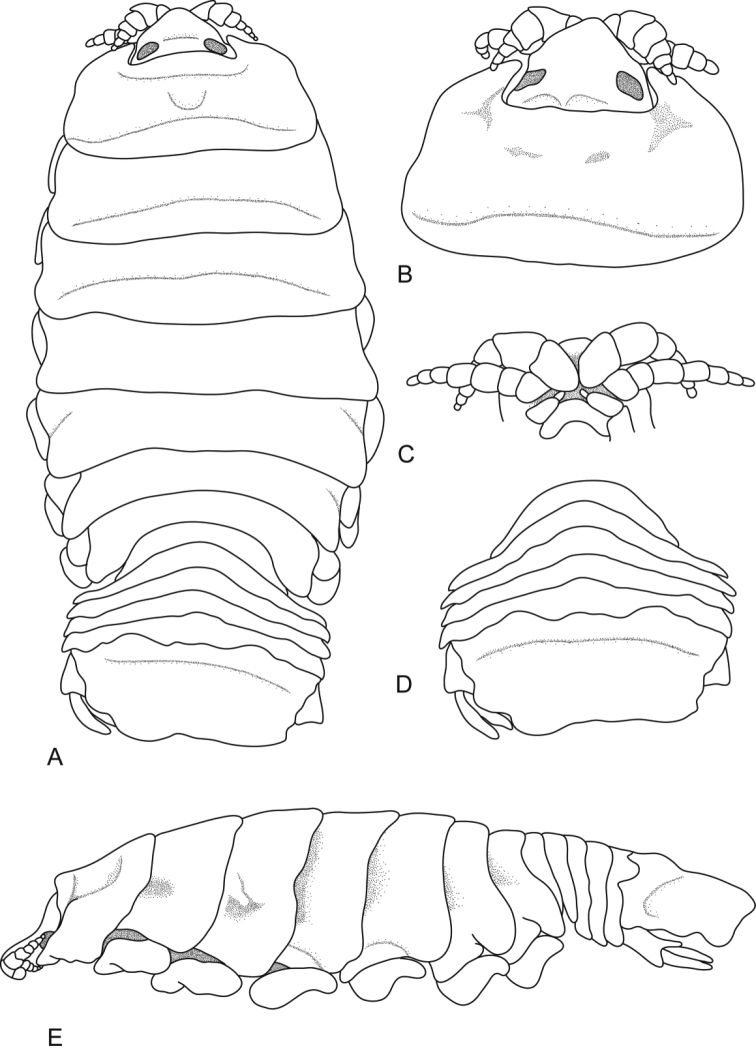
*Ceratothoa imbricata* (Fabricius, 1775), female (37 mm), (BMNH 1979.402.1 originally designated as holotype of *Ceratothoa banksii* Leach, 1818): **A** dorsal view **B** antero-dorsal view of pereonite 1 and cephalon **C** ventral view of cephalon **D** dorsal view of pleotelson **E** lateral view.

**Figure 18.**
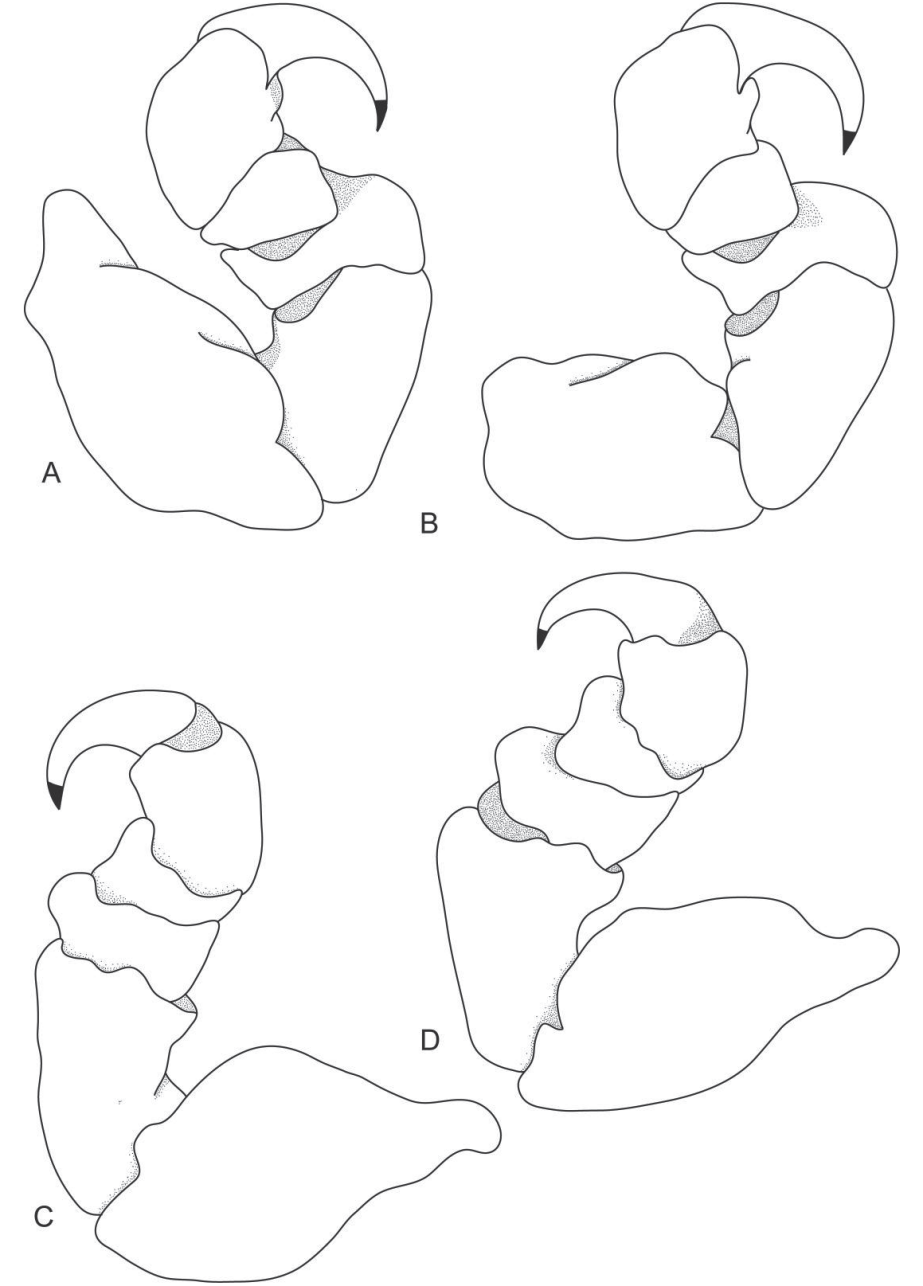
*Ceratothoa imbricata* (Fabricius, 1775), female (37 mm), (BMNH 1979.402.1 originally designated as holotype of *Ceratothoa banksii* Leach, 1818): **A** pereopod 1 **B** pereopod 2 **C** pereopod 6 **D** pereopod 7.

##### Distribution.

Australia (Schioedte and Meinert 1883, Miers 1884, Hale 1927, 1940), New Zealand (Fabricius 1775, 1793) and Indonesia (Schioedte and Meinert 1883).

Yu and Li (2003) included a figure of a specimen described as *Ceratothoa imbricata* from Chinese waters; the figures show that the antenna bases do not touch, which excludes the species from *Ceratothoa*.

##### Hosts.

From the mouth of a salmon–trout; from a *Monacanthus* sp. (Miers 1884); in the Australian jack mackerel, *Trachurus declivis* (Hale 1926, 1929); in snapper *Chrysophrys auratus* (previously *Pagrosomus auratus*), red gurnard (*Chelidonichthys kumu*), and mullet (*Mugil* sp.) (Hale 1926, 1929); in mouth of *Girella tricuspidata* (Hale 1926, 1929); trevally *Psuedocaranx dentex* (previously *Caranx georgianus*) (Hale 1926, 1929).

Schioedte and Meinert (1883) mention a fish they thought may be a “red hottentot (*Sargi hottentotti* Sm.??)” collected from the Cape of Good Hope in South Africa. This could refer to the red roman fish, *Chrysoblephus laticeps* (see Kensley 1978), the Zebra (*Diplodus cervinus hottentotus*) or the Hottentot (*Pachymetopon blochii*) as “*Sargi hottentotti*” is not a valid taxonomic name and cannot be found in current fish database searches. No fresh material or museum material of these specimens from the red roman or those collected by Kensley (1978) could be found so these records are not accepted.

##### Remarks.

*Ceratothoa imbricata* can be identified by a large pereonite 1 with anterolateral margins extending past the eyes; uropods as long or longer than the pleotelson margin; merus with bulbous protrusion; a blunt rostrum; and body widest at pereonite 5.

It is apparent that over the years there have been many misidentifications of *Ceratothoa imbricata*, *Ceratothoa banksii* and *Ceratothoa trigonocephala*, with these names being widely misapplied. The description of *Ceratothoa banksii* from New Zealand, given by Miers (1876) can also be applied to the small Australian *Ceratothoa imbricata* specimens of Miers (1884), with only some slight variations in eyes, smaller anterolateral extensions on pereonite, 1 and a slightly arched pleotelson posterior margin. The original description of *Ceratothoa banksii* by Leach (1818) also described the pleotelson as “nearly straight” but according to Miers (1884), desiccation had caused the specimen to roll slightly. Many authors agreed with the synonymy of *Ceratothoa banksii* with *Ceratothoa imbricata* including Stebbing (1893), Nierstrasz (1915) and Trilles (1973) and we maintain this synonymy, however this needs further investigation, especially when fresh material becomes available.

Trilles (1994) placed Hale’s (1926, 1927, 1929, 1940) records of *Ceratothoa imbricata* into synonymy with *Ceratothoa trigonocephala*. After reviewing Hale’s (1926) figures, we conclude that his original identification of *Ceratothoa imbricata* is correct.

No South African specimens were found, fresh or from museum collections, that could be identified as *Ceratothoa imbricata*, and the species is here excluded from the South Africa fauna.

#### Ceratothoa trigonocephala

(Leach, 1818)

http://species-id.net/wiki/Ceratothoa_trigonocephala

Figs 19 – 21

Cymothoa trigonocephala Leach, 1818: 353; Guérin-Méneville and Cuvier 1829–1843: 26, pl. 29, fig. 2; Milne Edwards 1840: 272–273; Ellis 1981: 124.
Ceratothoa trigonocephala . – Schioedte and Meinert 1883: 358–364, tab. XVI (Cym. XXIII) Figs 1–7.

##### Material examined.

Lectotype [here designated]: The Natural History Museum, London (NHMUK 2013.1013) – female specimen (42 mm TL) collected by W.E. Leach, White’s MS Cat no. 404 a, b, host and locality unknown. Also noted: the female drawn was very squashed and missing pereonite 1.

Paralectotype. The Natural History Museum, London (BMNH 1979.404.2) – female specimen (17 mm TL without cephalon) collected by W.E. Leach, White’s MS Cat no. 404 a, b, host and locality unknown. Also noted: damaged female, missing the cephalon and oostegites, with dissected uropods.

##### Description of lectotype.

Body margins sub-parallel, 2.4 times as long as greatest width, dorsal surfaces smooth and polished in appearance, widest at pereonite 5 and pereonite 6, most narrow at pereonite 1, lateral margins subparallel. Cephalon 0.6 times longer than wide, visible from dorsal view, triangular. Frontal margin rounded to form blunt rostrum. Eyes not visible. Pereonite 1 with slight indentations, anterior border slightly indented, anterolateral angle with distinct anterior projection, posterior margins of pereonites smooth and straight. Pereonites 1–5 increasing in length and width; 6–7 decreasing in length and width; 6 and 7 narrower. Pleon with pleonite 1 most narrow, visible in dorsal view; pleonites posterior margin smooth, mostly concave; posterolateral angles of pleonite 2 rounded, not posteriorly produced. Pleonites 3–5 similar in form to pleonite 2. Pleonite 5 with posterolateral angles free, not overlapped by lateral margins of pleonite 4, posterior margin produced medially. Pleotelson 0.5 times as long as anterior width, dorsal surface with lateral indent, lateral margins weakly convex, posterior margin sub-truncate, without median point. Antennule more stout than antenna, comprised of 7 articles. Antenna comprised of 7 articles. Pereopod 1 basis 1.4 times as long as greatest width; ischium 0.8 times as long as basis; merus proximal margin without bulbous protrusion; carpus with rounded proximal margin; propodus 1.4 times as long as wide; dactylus slender, 1.2 as long as propodus, 2.3 times as long as basal width. Pereopod 2 propodus 1.4 as long as wide; dactylus 1.1 as long as propodus. Pereopods 3 similar to pereopod 2. Pereopod 6 basis 1.5 times as long as greatest width, ischium 0.8 times as long as basis, propodus 1.5 as long as wide, dactylus 1.1 as long as propodus. Pereopod 7 basis 1.4 times as long as greatest width; ischium 0.8 as long as basis, without protrusions; merus proximal margin with slight bulbous protrusion, merus 0.4 as long as ischium, 0.7 times as long as wide; carpus 0.3 as long as ischium, without bulbous protrusion, 0.7 times as long as wide; propodus 0.6 as long as ischium, 1.6 times as long as wide; dactylus slender, 1.2 as long as propodus, 2.5 times as long as basal width. Uropod more than half the length of pleotelson, peduncle 0.7 times longer than rami, peduncle lateral margin without setae; rami not extending beyond pleotelson, marginal setae absent, apices narrowly rounded.

**Figure 19.**
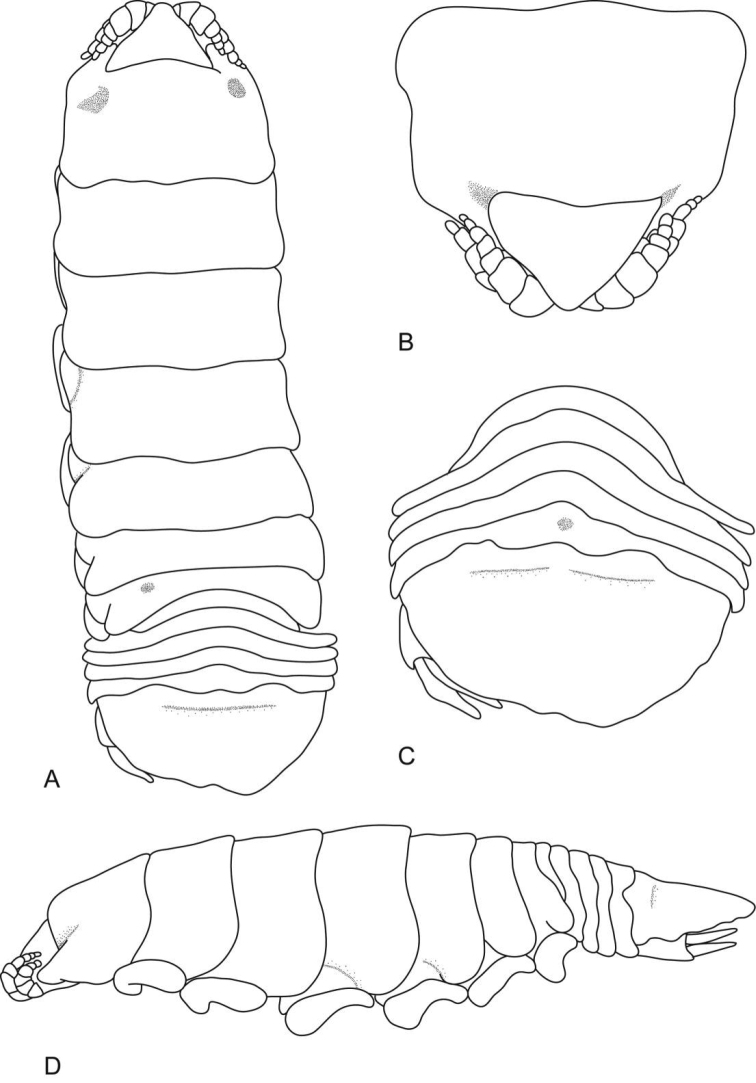
*Ceratothoa trigonocephala* (Leach, 1818), female lectotype (42 mm) (NHMUK 2013.1013): **A** dorsal view **B** antero-dorsal view of pereonite 1 and cephalon **C** dorsal view of pleotelson **D** lateral view.

**Figure 20.**
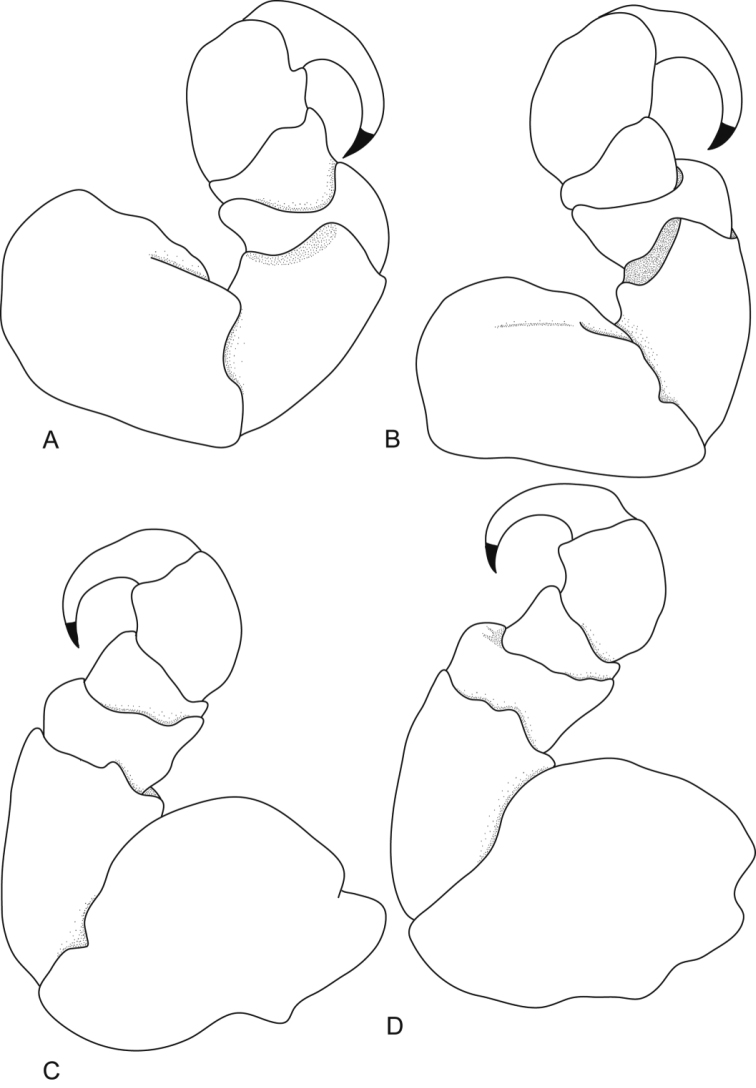
*Ceratothoa trigonocephala* (Leach, 1818), female lectotype (42 mm) (NHMUK 2013.1013): **A** pereopod 1 **B** pereopod 2 **C** pereopod 6 **D** pereopod 7.

##### Distribution.

Predominately the Indo-Pacific region: Australia; Vanuatu; and Indonesia (Schioedte and Meinert 1883), but given the uncertainty over the identity of these records the distribution remains entirely uncertain.

##### Hosts.

There are currently no confirmed hosts for this species.

##### Remarks.

*Ceratothoa trigonocephala* has a triangular cephalon, for which it is named, and arched carinae on the last pair of pereopods. It is identified by the subequal pereonites 1–4; mid-dorsal protrusion on pereonite 1; short and bluntly rounded anterolateral margins of pereonite 1; and uropods which do not extend past the pleotelson posterior margin. The type locality and host for *Ceratothoa trigonocephala* were not mentioned in the original work by Leach (1818).

Previously, Filhol (1885) noted that *Ceratothoa trigonocephala*, *Ceratothoa huttoni* Filhol, 1885 and *Ceratothoa novaezelandiae* Filhol, 1885 were three separate species based on small morphological differences. Some of these differences included the shape of the antennae; the shape and dimensions of the pereonites; and pigmentation. All three of these species were later combined into one as *Ceratothoa trigonocephala*, with the differences recognised as intraspecific and not interspecific (Trilles 1972). After reviewing the drawings, *Ceratothoa huttoni* was found to not resemble the *Ceratothoa trigonocephala* holotype and the identity of *Ceratothoa novaezelandiae* could not be confirmed and thus these synonymies are not upheld here.

*Ceratothoa trigonocephala* has often been confused with *Ceratothoa imbricata* and a complete redescription and species clarification on these two species was needed. Differences between *Ceratothoa imbricata* and *Ceratothoa trigonocephala*, based on description of the type specimens include: *Ceratothoa imbricata* pereonite 1 is larger than pereonites 2–4 while in *Ceratothoa trigonocephala* these four pereonites are subequal; the posterior margin of pereonite 1 is curved in *Ceratothoa imbricata* and straight in *Ceratothoa trigonocephala*; *Ceratothoa imbricata* had a bulbous protrusion on the merus of pereopod 1 which is absent in *Ceratothoa trigonocephala*; and the uropods of *Ceratothoa trigonocephala* are shorter than the pleotelson but are longer in *Ceratothoa imbricata*. Furthermore, *Ceratothoa imbricata* has a more narrow and produced anterolateral angles on pereonite 1; a more rounded anterior margin on the cephalon; longer uropods which extend to or past the posterior margin of the pleotelson; and pereonite 1 is longer than pereonites 2–4 which are almost subequal in *Ceratothoa trigonocephala*.

Although this species had been recorded from South Africa (Kensley 1978, 2001), no South African specimens were found during the present study that could be identified as *Ceratothoa trigonocephala*, and the species is here excluded from the South Africa fauna.

**Figure 21.**
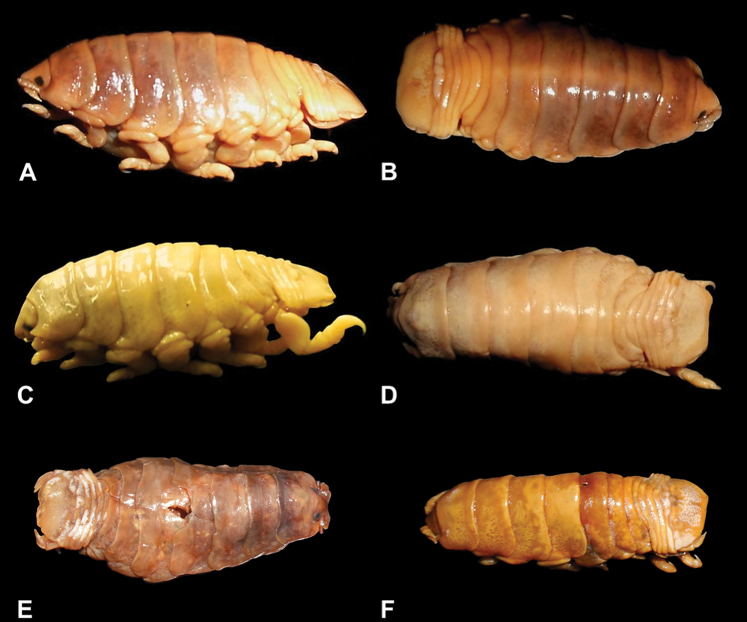
Photographs of the *Ceratothoa* specimens studied: **A** lateral view of *Ceratothoa africanae* sp. n. (SAM A45938) **B** dorsal view of *Ceratothoa africanae* sp. n. (SAM A45938) **C** lateral view of *Ceratothoa famosa* sp. n. (SAM A45941) **D** dorsal view of *Ceratothoa famosa* sp. n. (SAM A45941) **E** dorsal view of *Ceratothoa imbricata* (Fabricius, 1775) (BMNH 1979.403.1) **F** dorsal view of *Ceratothoa trigonocephala* (Leach, 1818) (NHMUK 2013.1013).

## Conclusion

We regard *Ceratothoa imbricata* and *Ceratothoa trigonocephala* as valid and distinct species despite the historical confusion over their respective identities. When comparing the holotype of *Ceratothoa banksii* to *Ceratothoa imbricata*, a number of similarities and differences could be seen but without other new material these differences seemed insufficient to remove the synonymy at present.

Records of *Ceratothoa imbricata* and *Ceratothoa trigonocephala* without figures or mention of museum material are impossible to verify. The synonymy presented here includes only those records that we can confirm against our redescription of the type material.

Although valid species, *Ceratothoa imbricata* and *Ceratothoa trigonocephala* do not occur in South Africa. These misidentifications were most probably referring to one of the two new species, *Ceratothoa africanae* sp. n. or *Ceratothoa famosa* sp. n.

## Supplementary Material

XML Treatment for Ceratothoa

XML Treatment for Ceratothoa retusa

XML Treatment for Ceratothoa africanae

XML Treatment for Ceratothoa famosa

XML Treatment for Ceratothoa imbricata

XML Treatment for Ceratothoa trigonocephala
